# Dual Expression Profile of Type VI Secretion System Immunity Genes Protects Pandemic *Vibrio cholerae*

**DOI:** 10.1371/journal.ppat.1003752

**Published:** 2013-12-05

**Authors:** Sarah T. Miyata, Daniel Unterweger, Sydney P. Rudko, Stefan Pukatzki

**Affiliations:** Department of Medical Microbiology and Immunology, University of Alberta, Edmonton, Alberta, Canada; University of Washington, United States of America

## Abstract

The *Vibrio cholerae* type VI secretion system (T6SS) assembles as a molecular syringe that injects toxic protein effectors into both eukaryotic and prokaryotic cells. We previously reported that the *V. cholerae* O37 serogroup strain V52 maintains a constitutively active T6SS to kill other Gram-negative bacteria while being immune to attack by kin bacteria. The pandemic O1 El Tor *V. cholerae* strain C6706 is T6SS-silent under laboratory conditions as it does not produce T6SS structural components and effectors, and fails to kill *Escherichia coli* prey. Yet, C6706 exhibits full resistance when approached by T6SS-active V52. These findings suggested that an active T6SS is not required for immunity against T6SS-mediated virulence. Here, we describe a dual expression profile of the T6SS immunity protein-encoding genes *tsiV1*, *tsiV2*, and *tsiV3* that provides pandemic *V. cholerae* strains with T6SS immunity and allows T6SS-silent strains to maintain immunity against attacks by T6SS-active bacterial neighbors. The dual expression profile allows transcription of the three genes encoding immunity proteins independently of other T6SS proteins encoded within the same operon. One of these immunity proteins, TsiV2, protects against the T6SS effector VasX which is encoded immediately upstream of *tsiV2*. VasX is a secreted, lipid-binding protein that we previously characterized with respect to T6SS-mediated virulence towards the social amoeba *Dictyostelium discoideum*. Our data suggest the presence of an internal promoter in the open reading frame of *vasX* that drives expression of the downstream gene *tsiV2*. Furthermore, VasX is shown to act in conjunction with VasW, an accessory protein to VasX, to compromise the inner membrane of prokaryotic target cells. The dual regulatory profile of the T6SS immunity protein-encoding genes *tsiV1*, *tsiV2*, and *tsiV3* permits *V. cholerae* to tightly control T6SS gene expression while maintaining immunity to T6SS activity.

## Author Summary

*Vibrio cholerae* is the causative agent of the diarrheal disease cholera. This bacterium uses the type VI secretion system (T6SS) to kill other bacteria and host cells. The T6SS is a molecular syringe that Gram-negative bacteria use to inject toxic effectors into target cells in a contact-dependent manner. The *V. cholerae* T6SS secretes at least three distinct effectors, VasX, TseL, and VgrG-3 to confer antimicrobial activity. To protect itself from an oncoming attack by neighboring bacteria, *V. cholerae* produces three immunity proteins, TsiV1, TsiV2, and TsiV3 that specifically inactivate the activity of their respective effectors. We determined that the genes encoding TsiV1, TsiV2, and TsiV3 are controlled in a dual fashion that ensures expression of these genes at all times. This provides *V. cholerae* with constant protection from a T6SS attack by nearby close relatives. Thus, the T6SS gene cluster is a toxin/immunity system that can both kill and protect bacterial cells. Here, we characterize the mechanism of one T6SS effector, VasX, that disrupts the inner membrane of susceptible bacteria. The immunity protein TsiV2 protects prokaryotic cells against VasX-mediated toxicity.

## Introduction

*Vibrio cholerae* is the etiological agent of the diarrheal disease cholera. This pathogen utilizes a wide array of virulence factors during host infection including the well-characterized cholera toxin (CT) and toxin-coregulated pilus (TCP). In addition, *V. cholerae* possesses numerous other virulence factors including the type VI secretion system (T6SS), a recently described mechanism used by numerous Gram-negative bacteria to export effectors across their cell envelopes. In contrast to TCP and CT, whose presence is restricted to a subset of virulent *V. cholerae* strains, the *V. cholerae* T6SS is highly conserved and is present in strains of all serogroups sequenced to date. Structurally, the T6SS resembles an inverted bacteriophage; it assembles in the *V. cholerae* cytoplasm and docks onto a baseplate complex situated in the bacterial envelope. Two alleles on the small and large chromosome encode hemolysin-coregulated protein (Hcp) [1], which polymerizes [2] and forms the inner tube of the T6SS injectosome and acts as a chaperone for T6SS effectors [3]. Contraction of the outer sheath (formed by VipA and VipB) around the formed Hcp nanotube leads to the ejection of the Hcp tube decorated with a VgrG trimer consisting of three different VgrG proteins: VgrG-1, VgrG-2, and VgrG-3 [4]–[10]. VgrG-1 and VgrG-3 carry enzymatic C-terminal extensions that crosslink actin or degrade the peptidoglycan layer, respectively, upon translocation into target cells [4], [6], [10]–[12]. ATP hydrolysis by the inner membrane protein VasK provides the energy for Hcp secretion [13]. A recent report suggests that proteins from the PAAR (proline-alanine-alanine-arginine) repeat superfamily form a sharp conical extension on the VgrG cap. This extension is able to load additional T6SS effectors besides the VgrGs, which are then delivered simultaneously into target cells in a single contraction-driven translocation event [14].

The O37 serogroup *V. cholerae* strain V52 [15] constitutively synthesizes T6SS structural proteins and effectors and actively engages in T6SS-mediated virulence under standard laboratory conditions [4], [5], [16]. Conversely, the pandemic-causing O1 serogroup strain C6706 possesses a full complement of T6SS-encoding genes, but does not express T6SS genes encoding structural apparatus components and effectors under laboratory conditions. Null-mutations in the genes encoding the quorum sensing regulator LuxO and the global regulator TsrA lead to production and secretion of the T6SS protein Hcp, and T6SS-mediated virulence in *V. cholerae* strain C6706, demonstrating that this pandemic strain employs a tightly controlled, fully active T6SS [17].

**Figure 1:**
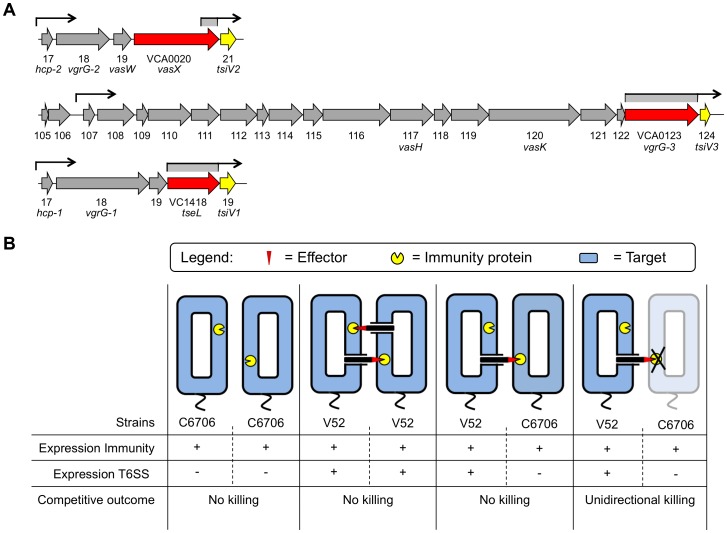
Dual regulatory profile of T6SS immunity protein-encoding genes. (A) Schematic representation of the *V. cholerae* T6SS gene clusters. Toxin-encoding genes are shown in red and immunity protein-encoding genes in yellow. Promoters upstream of the individual T6SS clusters are indicated by arrows. Regions with promoter activity that drive expression of immunity protein-encoding genes are indicated in grey below arrows. (B) Two *V. cholerae* strains, C6706 and V52, exemplify the dual regulatory profile of the T6SS system. In the T6SS-off state, C6706 expresses only the T6SS immunity proteins (first panel). In contrast, V52 expresses structural, effector, and immunity T6SS proteins, the latter of which provide protection from T6SS-active kin bacteria (second panel). When T6SS-active V52 come in contact with C6706 in the T6SS-off state, C6706 is protected from a T6SS-mediated attack without engaging in T6SS-mediated virulence (third panel). C6706 succumbs to killing by V52 when immunity genes are removed or not expressed (fourth panel).

The *V. cholerae* T6SS is encoded by three separate gene clusters [5] (Figure 1A). One source of T6SS regulation, VasH (encoded by VCA0117), is encoded in all *V. cholerae* genomes sequenced so far and acts as an activator of the alternate sigma factor 54. A regulatory role for VasH in the expression of genes within the two auxiliary T6SS gene clusters encoding *(i)* Hcp-1 and VgrG-1, and *(ii)* VgrG-2 and VasX has been established in *V. cholerae* El Tor and classical O1, and non-O1/non-O139 strains [18]–[20].

T6SSs mediate toxicity towards both eukaryotes and prokaryotes [4], [5], [16], [21]–[32]. The *V. cholerae* T6SS mediates virulence towards murine macrophages and the amoeboid host *Dictyostelium discoideum* [4], [5], [16], [18], [21] as well as a variety of Gram-negative bacteria including *Escherichia coli*, *Salmonella* Typhimurium, and *Citrobacter rodentium* [16], [33]. Importantly, V52 bacteria do not kill each other, and some bacteria such as *V. cholerae* C6706 are resistant to killing by V52 [16]. Although all sequenced *V. cholerae* strains possess a full complement of T6SS-encoding genes, only some strains are known to express these genes under laboratory conditions. V52 has a constitutively active T6SS and produces hallmark T6SS proteins such as Hcp and VgrGs when grown in liquid culture [5]. In contrast, C6706 maintains a T6SS-silent state and does not produce Hcp and VgrGs under standard laboratory conditions [34]; yet C6706 is immune to killing by V52 [11], [16].

T6SS-mediated killing of prokaryotes has been reported in other bacterial species including *Pseudomonas aeruginosa*, *Serratia marcescens*, and *Burkholderia thailandensis* [23], [25], [26], [35], [36]. The *P. aeruginosa* T6SS encodes effectors as part of a toxin/antitoxin (TA) gene system in which the effector (toxin) is injected into the target cell where it degrades the peptidoglycan in the target cell unless inhibited by an immunity protein (antitoxin) [35].

TA systems are not restricted to T6SS gene clusters, and one other notable TA system subset are colicins—small secreted peptides produced by and toxic to certain strains of *E. coli* [37]. Colicins employ a diverse range of mechanisms to kill target cells, including degradation of RNA and DNA [38]–[44], inhibition of murein synthesis [45], and pore-formation in the inner membrane [46]–[49]. In the latter case, the pore in the inner membrane forms a voltage-dependent ion channel that dissipates membrane potential and halts cellular respiration [48]–[55]. Pore-formation in the inner membrane can occur only if colicin is presented to the target cell from the periplasmic face [56], [57]. Consequently, cytoplasmic production of colicins is not toxic to the producing cell [57].

We previously identified the protein VasX and characterized this T6SS effector with respect to its role in T6SS-mediated killing of *D. discoideum* [21]. In this study, we characterize the mechanism by which VasX acts as a bacterial effector. We also present evidence that VasW, the product of the gene directly upstream of *vasX*, is necessary for VasX-mediated bacterial killing. By screening a C6706 T6SS transposon library [58], we identified the VasX immunity protein-encoding gene VCA0021 located directly downstream of *vasX*, and two other T6SS-encoding genes, VCA0124 and VC1419, that encode additional immunity proteins. We demonstrate that all three immunity protein-encoding genes are controlled in a dual regulatory fashion, ensuring that even T6SS-silent strains such as C6706 are protected from T6SS-mediated killing by neighboring bacterial competitors (Figure 1B).

## Materials and Methods

### Bacterial strains and growth conditions

The strains and plasmids used in this study are listed in Table 1 and Table 2, respectively. A streptomycin-resistant *V. cholerae* strain V52 (O37 serogroup) lacking *hapA*, *rtxA*, and *hlyA* genes was used as a T6SS-active strain in all experiments in this study unless otherwise noted in the figure legend. A streptomycin- and rifampicin-resistant *V. cholerae* strain C6706 (O1 serogroup) was used as a T6SS-silent strain in all experiments. DH5α λpir and SM10 λpir were used for cloning, and mating of pWM91-based plasmids, respectively. Unless stated otherwise, bacteria were grown in Luria-Bertani (LB) broth at 37°C with shaking (200 rpm). Antibiotic concentrations used were 100 µg·mL^−1^ ampicillin, 10 µg·mL^−1^ chloramphenicol, 100 µg·mL^−1^ streptomycin, 50 µg·mL^−1^ kanamycin, and 50 µg·mL^−1^ rifampicin. Arabinose was added at a final concentration of 0.1% to induce expression from the P_BAD_ promoter.

**Table 1:** Bacterial strains.

Strain	Description	Reference or source
*Vibrio cholerae*, V52	O37 serogroup strain, sm^R^	Zinnaka *et al.*, 1972
*Vibrio cholerae*, V52 HRH	O37 serogroup strain, Δ*hapA*, Δ*rtxA*, Δ*hlyA*, sm^R^	Pukatzki *et al.*, 2006
*Vibrio cholerae*, V52Δ*vasK*	V52 mutant lacking *vasK* (VCA0120)	Pukatzki *et al.*, 2006
*Vibrio cholerae*, V52Δ*vasX*	V52 mutant lacking *vasX* (VCA0020)	Miyata *et al.*, 2010
*Vibrio cholerae*, V52Δ*vasH*	V52 mutant lacking *vasH* (VCA0117)	Kitaoka *et al.*, 2011
*Vibrio cholerae*, V52Δ*vasW*	V52 mutant lacking *vasW* (VCA0019)	This study
*Vibrio cholerae*, V52Δ*vgrG-3*	V52 mutant lacking *vgrG-3* (VCA0123)	Pukatzki *et al.*, 2006
*Vibrio cholerae*, V52Δ*tseL*	V52 mutant lacking *tseL* (VC1418)	This study
*Vibrio cholerae*, V52Δ*tseL*Δ*vgrG-3*	V52 mutant lacking *tseL* (VC1418) and *vgrG-3* (VCA0123)	This study
*Vibrio cholerae*, V52Δ*tseL*Δ*vgrG-3*Δ*vasX*	V52 mutant lacking *tseL* (VC1418), *vgrG-3* (VCA0123), and *vasX* (VCA0020)	This study
*Vibrio cholerae*, V52Δ*vgrG*-3Δ*vasX*	V52 mutant lacking *vgrG-3* (VCA0123) and *vasX* (VCA0020)	This study
*Vibrio cholerae*, V52Δ*vgrG*-3Δ*vasW*	V52 mutant lacking *vgrG-3* (VCA0123) and *vasW* (VCA0019)	This study
*Vibrio cholerae*, V52Δ*vasH*Δ*vasX*	V52 mutant lacking *vasH* (VCA0117) and *vasX* (VCA0020)	This study
*Vibrio cholerae*, C6706	O1 El Tor, Sm^R^, Rif^R^	Dr. J. Mekalanos, Harvard Medical School
*Vibrio cholerae*, C6706Δ*tsiV1*	C6706 mutant lacking *tsiV2* (VC1419)	This study
*Vibrio cholerae*, C6706Δ*tsiV2*	C6706 mutant lacking *tsiV2* (VCA0021)	This study
*Vibrio cholerae*, C6706Δ*tsiV3*	C6706 mutant lacking *tsiV3* (VCA0124)	This study
*Vibrio cholerae*, C6706Δ*vasX*	C6706 mutant lacking *vasX* (VCA0020)	This study
*Vibrio cholerae*, C6706Δ*vgrG-3*	C6706 mutant lacking *vgrG-3* (VCA0123)	This study
*Vibrio cholerae*, C6706Δ*tseL*	C6706 mutant lacking *tseL* (VC1418)	This study
*Vibrio cholerae*, NIH41^w^	O1 Classical, Sm^R^, amp^R^, *lacZ::pJL1*	This study
*Vibrio cholerae*, O395^w^	O1 Classical, Sm^R^, amp^R^, *lacZ::pJL1*	This study
*Vibrio cholerae*, MAK 757^w^	O1 El Tor, Sm^R^, amp^R^, *lacZ::pJL1*	This study
*Vibrio cholerae*, C6709^w^	O1 El Tor, Sm^R^, amp^R^, *lacZ::pJL1*	This study
*Vibrio cholerae*, N16961^w^	O1 El Tor, Sm^R^, amp^R^, *lacZ::pJL1*	This study
*Vibrio parahaemolyticus* RIMD2210633	Rif^R^	Dr. N. Thomas, Dalhousie University
*Escherichia coli*, DH5α λpir	fhuA2 D(argF-lacZ)U169 phoA glnV44 W80 D(lacZ)M15 gyrA96 recA1 relA1 endA1 thi-1 hsdR17	Dr. D. Provenzano University of Texas at Brownsville
*Escherichia coli*, SM10 λpir	KmR, thi-1, thr, leu, tonA, lacY, supE, recA::RP4-2-Tc::Mu, pir	Dr. J. Mekalanos, Harvard Medical School
*Escherichia coli*, Top10	F- mcrA Δ(mrr-hsdRMS-mcrBC) φ80lacZΔM15 ΔlacX74 nupG recA1 araD139 Δ(ara-leu)7697 galE15 galK16 rpsL(Str^R^) endA1 λ^−^	Invitrogen; Carlsbad, CA
*Escherichia coli*, MG1655	F- lambda- ilvG- rfb-50 rph-1, Rif^R^	Dr. T. Raivio, University of Alberta

**Table 2:** Plasmids.

Plasmid	Description	Reference
pBAD24	pBAD vector, pBR322 ori, araC, Amp^R^	Guzman et al., 1995
pBAD24-vasX	pBAD24 carrying *vasX* (VCA0020) of the *V. cholerae* strain V52	Miyata et al., 2010
pBAD24-vasX::FLAG	pBAD24 carrying *vasX* from V52 with C-terminal FLAG tag	This study
pBAD24-tsiV2::FLAG	pBAD24 carrying *tsiV2* (VCA0021)from V52 with C-terminal FLAG tag	This study
pBAD24-LS	pBAD24 with periplasmic targeting sequence	Dr. J. Weiner, UAlberya
pBAD24-LS::vasX	pBAD24-LS carrying *vasX* from V52	This study
pBAD24-LS::core	pBAD24-LS carrying *vgrG-3* core (lacks C-terminal enzymatic domain)	This study
pBAD24-vasW::FLAG	pBAD24 carrying *vasW* (VCA0019) of the *V. cholerae* strain V52	This study
pBAD24-tsiV1::FLAG	pBAD24 carrying *tsiV1* (VC1419) from V52 with C-terminal FLAG tag	This study
pBAD24-tsiV3::FLAG	pBAD24 carrying *tsiV3* (VCA0124)from V52 with C-terminal FLAG tag	Brooks et al., 2013
pBAD33	pBAD vector, pACYC184 ori, araC, Cm^R^	Guzman et al., 1995
pBAD33-*tsiV2::6xHis*	pBAD33 carrying *tsiV2* with C-terminal hexa-histidine tag	This study
pAH6	pAH6 vector, pACYC184 ori, Cm^R^	Hsaio et al., 2006
pAH6-P_hcp_	pAH6 carrying *hcp-2* promoter (−1 to −400)	This study
pAH6-vasX	pAH6 carrying *vasX* from *V. cholerae* V52 (lacking start codon)	This study
pAH6-vasX(1-1345)	pAH6 carrying *vasX* (nucleotides 4-1345) from V52	This study
pAH6-vasX(1575-3258)	pAH6 carrying *vasX* (nucleotides 1575-3258) from V52	This study
pAH6-vasX(2208-3258)	pAH6 carrying *vasX* (nucleotides 2208-3258) from V52	This study
pAH6-vasX(3006-3258)	pAH6 carrying *vasX* (nucleotides 3006-3258) from V52	This study
pAH6-tseL	pAH6 carrying *tseL* from *V. cholerae* V52 (lacking start codon)	This study
pAH6-vgrG-3	pAH6 carrying *vgrG-3* from *V. cholerae* V52 (lacking start codon)	This study
pWM91	oriR6K mobRP4 lacI ptac tnp mini-Tn10Km; Km^R^ Amp^R^	Metcalf et al., 1996
pRK2013	Col E1 rep, *tra* ^+^ *mob* ^+^, Kan^R^	Clontech
pDONR221-*tsiV1*	Col E1 rep, Kan^R^	Harvard Institute of Proteomics
pDONR221-*tsiV2*	Col E1 rep, Kan^R^	Harvard Institute of Proteomics
pDONR221-*tsiV3*	Col E1 rep, Kan^R^	Harvard Institute of Proteomics
pJET1.2/blunt	Vector for cloning blunt ended PCR products, Amp^R^	Thermo Fisher Scientific
pCR2.1-TOPO TA	Vector for cloning PCR products, Amp^R^	Invitrogen
pJL1	Suicide plasmid for allele exchange in *V. cholerae lacZ* Ori6k, mob^+^, amp^R^	Dr. D. Provenzano, University of Texas at Brownsville

### In-frame deletions and plasmid construction

In-frame deletion of T6SS-encoding genes was performed as described previously [59]. Construction of the *vasX*, *vasH*, and *vasK* knockout constructs was also previously described [5], [18], [21]. *VasW*, *vgrG-3*, *tseL*, *tsiV1*, *tsiV2*, and *tsiV3* knockout constructs were created using the primers listed in Table 3. PCR products resulting from primer combinations A/B and C/D were stitched together by overlapping PCR. The resulting knockout construct was digested with *Bam*HI and cloned into the suicide plasmid pWM91 [59]. V52Δ*vgrG-3* [5] was used as the parent strain in which *vasW* was deleted to create the V52Δ*vgrG-3*Δ*vasW* double mutant. V52Δ*vasH* was used as the parent strain in which *vasX* was deleted to create the V52Δ*vasH*Δ*vasX* double mutant.

**Table 3:** Oligonucleotide sequences.

Primer name	Destination plasmid	Direction^a^	Sequence^b^
5′ *vasX*	pBAD24	F	GGTACC CATGAGTAATCCCAAT
3′ *vasX::FLAG*		R	TCTAGA TTATTTATCATCATCATCTTTATAATCACCTTTTCCTACAACGAG
5′ *vasX*	pAH6	F	AAGCTT AGTAATCCCAATCAAGCTGCG
3′ *vasX*		R	TCTAGA TTAACCTTTTCCTACAAC
3′ *vasX* (1345)		R	TCTAGA ACGCTTGCTGAACCTCATCT
5′ *vasX* (1575)		F	AAGCTT TGCGACCGCAATCGCTAACT
5′ *vasX* (2208)		F	AAGCTT CCGCTATAAGTCGCACAAT
5′ *vasX*(3006)		F	AAGCTT GGCGGTAACACCAATACTCA
5′ P_hcp_		F	AAGCTT GCTCTTCCCGTTTGTCGTTATATAC
3′ P_hcp_		R	TCTAGA GGCTATTTCCTTTCAATAAATC
5′ *tseL*		F	TCTAGAGATTCATTTAATTATTGC
3′ *tseL*		R	GTCGACTCATCTTATTTGCACCTTG
5′ *vgrG-3*		F	TCTAGAGCAAGGTTACAGTTTCAATTA
3′ *vgrG-3*		R	TCTAGATCATTTTATATCAACCTCCAAAC
5′ *vasX*	pBAD24-LS	F	TCTAGA AGTAATCCCAATCAAGCTGCG
3′ *vasX*		R	AAGCTT TTAACCTTTTCCTACAAC
5′ *vasW*	pBAD24	F	GAATTC ATGCGTTCAACAAATTCC
3′ *vasW::FLAG*		R	TCTAGA TCATTTATCATCATCATCTTTATAATCTCCTTGTACCTCCTGT
5′ *tsiV2*	pBAD33		AAGCTT GATGTTAATTGATAAAAATGAG
3′ *tsiV2::6xHis*		R	TCTAGA TTAGTCTTTTAATTCTTG
5′ pBAD-DEST49	pBAD24	F	GGTACC AAGTTTGTACAAAAAAGCTGAAC
3′ pBAD-DEST49::FLAG		R	TCTAGATCATTTATCATCATCATCTTTATAATCCACCACTTTGTACAAGAAAGCTG
k/o-*vasW* A	pWM91	F	GGATCC ATCGGTCATGAAG
k/o *vasW* B		R	TTACTCATCCTTGTACCTCATTTGTTGAACGCATTAAT
k/o *vasW* C		F	ATTAATGCGTTCAACAAATGAGGTACAAGGATGAGTAA
k/o *vasW* D		R	GGATCC CATTCAGCGCCAC
k/o *tseL* A		F	GGATCC CGTTTAAACAGGCGGTGGCG
k/o *tseL* B		R	GATAACCATGATTTCACAGCAAACCTTACC
k/o *tseL* C		F	GCTGTGAAATCATGGTTATCCCCTTAGTTC
k/o *tseL* D		R	GGATCC CACCGGCATTAATTATCATCAGATACC
k/o *vgrG-3* A		F	GGATCC CCACAAGTGAGCGTGCG
k/o *vgrG-3* B		R	TTATTCATTTTATATCAACCTGTAACCTTGCCATGCTG
k/o *vgrG-3* C		F	CAGCATGGCAAGGTTACAGGTTGATATAAAATGAATAA
k/o *vgrG-3* D		R	GGATCC GTAATGAAGATTTGATGAGG
k/o *tsiV1* A		F	GGATCC GCCATAGCTTAGGGGGCGC
k/o *tsiV1* B		R	GGCATTAATTATCATCAGAATTCAATAACTTCATCTTATTTGC
k/o *tsiV1* C		F	TAAGATGAAGTTATTGAATTCTGATGATAATTAATGCC
k/o *tsiV1* D		R	GGATCC ACACCTGCATCCTTAGCGCG
k/o *tsiV2* A		F	GGATC CAACCGATCTTGAAC
k/o *tsiV2* B		R	TGAGCTATTCCTCTTTTAATTTATCAATTAACATTTAA
k/o *tsiV2* C		F	TTAAATGTTAATTGATAAATTAAAAGAGGAATAGCTCA
k/o *tsiV2* D		R	GGATCC CTTATCTACTCGTTA
k/o *tsiV3* A		F	GGATCC AGCATTGGCGCTGTT
k/o *tsiV3* B		R	AATCCTAACTATTATCAACAAGCAAGTTATTCATTTTA
k/o *tsiV3* C		F	TAAAATGAATAACTTGCTTGTTGATAATAGTTAGGATT
k/o *tsiV3* D		R	GGATCC AGCGCGAGATCAATAC

a F, forward; R, reverse;

b Restriction sites are underlined.

Construction of plasmid pBAD24-*vasX* was described previously [21]. *VasX::FLAG* and *vasW::FLAG* were PCR-amplified using the primers listed in Table 3 with Accuprime Pfx DNA polymerase (Invitrogen) and cloned into pCR2.1 TOPO TA (Invitrogen) following 10 minute incubation at 72°C with TopTaq polymerase (Qiagen) to add 3′ adenine overhangs. *VasX::FLAG* was excised using *Kpn*I and *Xba*I and subcloned into pBAD24. *VasW::FLAG* was excised using *Eco*RI and *Xba*I and subcloned into pBAD24 [60].

The VasX immunity protein-encoding gene *tsiV2* was PCR amplified with a C-terminal 6×His tag using Phusion Taq (Thermo Fisher) and ligated into pJET1.2/blunt (Thermo Fisher). The fragment was excised with *Kpn*I and *Xba*I restriction enzymes, subcloned into pBAD33 [60], and transformed into *E. coli* Top10 (Invitrogen). A triparental mating technique [61] was used to introduce pBAD33-*tsiV2::6×His* into *V. parahaemolyticus* RIMD2210633. Top10/pBAD33-*tsiV2::6×His* (Cm^R^), *V. parahaemolyticus* (Rif^R^), and DH5α/pRK2013 (helper plasmid containing *tra* and *mob* genes) were mixed at a 1∶1∶1 ratio, spotted onto nonselective LB plates, and incubated for 6 hours at 37°C. The spot was harvested into 1 mL LB and pelleted at 13,000 rpm for 5 minutes. Cells were resuspended in 100 µL LB and plated on thiosulfate-citrate-bile salts-sucrose (TCBS) agar plates containing 50 µg·mL^−1^ rifampicin and 10 µg·mL^−1^ chloramphenicol to select for *V. parahaemolyticus*/pBAD33-tsiV2::6×His. Plates were incubated overnight at 37°C. Resulting colonies were restreaked onto TCBSrif^50^chlor^10^ to exclude false-positive transformants.

Construction of the periplasmic expression vectors pBAD24-LS and pBAD24-SecP::core was described previously [12]. The *vasX* gene was cloned in-frame with the sequence encoding a sec signal peptide (SecP) using *Xba*I and *Hind*III restriction sites. The resulting construct was transformed into *E. coli* Top10 (Invitrogen) for expression analysis and then into the indicated *Vibrio* strains.

Plasmid pAH6 [62] was a generous gift from Dr. Jun Zhu (University of Pennsylvania School of Medicine) and contains a promoter-less *lacZ* gene. *VasX* (4-3258, 4-1345, 1575-3258, 2208-3258), *tseL*, *vgrG-3*, and P_hcp_ were amplified using Phusion Taq (Thermo Fisher), cloned into the blunt-ended cloning vector pJET1.2/blunt (Thermo Fisher), and transformed into *E. coli* Top10 (Invitrogen). All *vasX* constructs, P_hcp_, and *tseL* were excised using *Xba*I and *Hind*III restriction sites and subcloned into pAH6. *VgrG-3* was excised using the *Xba*I restriction site and subcloned into pAH6. The resulting constructs were transformed into *E. coli* Top10, followed by *V. cholerae* V52, V52Δ*vasH*, and C6706.

Plasmids pBAD24-tsiV1::FLAG, pBAD24-tsiV2::FLAG, and pBAD24-tsiV3::FLAG were created using the Gateway recombination system. Briefly, the LR recombination site, including the *ccdB* negative selection sequence, was amplified out of pBAD-DEST-49 using the primers listed in Table 2 (forward primer adds a *Kpn*I site, reverse primer adds a stop codon, *Xba*I site, and FLAG epitope tag sequence). The PCR product was digested with *Xba*I and *Kpn*I and ligated into the corresponding sites of pBAD24 to create pBAD24gw::FLAG. The Gateway recombination reaction between pDONR221-*tsiV2* (Harvard Institute of Proteomics) and pBAD24gw::FLAG was performed according to the manufacturer's protocol (Invitrogen, Carlsbad, CA). The resulting plasmids were transformed into *E. coli* Top10, followed by transformation into *V. cholerae* C6706Δ*tsiV1*, C6706Δ*tsiV2*, C6706Δ*tsiV3*, C6706Δ*vasX*, C6706Δ*tseL*, or C6706Δ*vgrG-3*.

### Bioinformatics and statistical analyses

Bioinformatics were performed using BLASTn (nucleotide) and BLASTp (protein) algorithms (http://blast.ncbi.nlm.nih.gov/Blast.cgi). Statistical significance was determined using the Student's one-tailed, paired t-test with a significance cut-off of p<0.05.

### Bacterial killing assay

Killing assays were performed as described previously [16]. Briefly, bacterial strains were grown overnight on selective LB plates and resuspended in LB broth (or LB 3% NaCl for *V. parahaemolyticus*). Predator and prey were mixed at a 10∶1 ratio and spotted onto prewarmed LB agar plates and incubated at 37°C for 4 hours. Spots were harvested, serially diluted, and 10 µl of each dilution was spotted onto a selective LB plate. Plates were incubated overnight at 37°C and surviving prey (CFU·mL^−1^) were enumerated. The competitive index was calculated by dividing prey CFUs after exposure to V52 by prey CFUs following exposure to V52Δ*vasK*.

### Cytoplasmic production of VasX in C6706ΔtsiV2

An overnight culture of C6706Δ*tsiV2*/pBAD24-vasX was back-diluted 1∶100 in selective LB broth in the presence or absence of 0.1% arabinose to induce expression of *vasX*. Strains were grown in a 96 well plate on a Heidolph Titromax 1000 vibrating shaker at 900 rpm. OD_600_ readings were taken every 30 min for 8 h using a BioRad XMark microplate spectrophotometer. At the 7-hour time point, a cell lysate sample was collected from each sample, mixed with sodium dodecyl sulfate (SDS) protein sample buffer, and boiled for 10 min. The protein samples were subjected to SDS-PAGE followed by western blotting to detect VasX and DnaK.

### β-galactosidase assay

The OD_600_ was measured of overnight liquid cultures diluted 1/10 in Z buffer (0.06 M Na_2_HPO_4_, 0.04 M NaH_2_PO_4_, 0.01 M KCl, 0.001 M MgSO_4_, pH 7.0) containing 2.7% β-mercaptoethanol. To lyse bacteria, 10 µL of 0.1% SDS and 20 µL of chloroform were added and the mixture was vortexed for 5 sec. Tubes were incubated in a 28°C waterbath for 10 min, followed by addition of 200 µL *ortho*-nitrophenyl-β-galactoside (ONPG) buffer (4 mg·mL^−1^ ONPG dissolved in Z buffer using a sonicating waterbath). The time required for development of a yellow color was recorded and the reaction was halted by the addition of 500 µL of 1 M Na_2_CO_3_. The OD_420_ of each sample was measured using a BioRad XMark microplate spectrophotometer. Miller units were calculated based on the equation (OD_420_/(OD_600_·time·volume)×10^3^).

Strains V52, C6706, and V52Δ*vasH* used in this assay each possess a chromosomal copy of *lacZ*. Strains O395^w^, NIH41^w^, MAK757^w^, C6709^w^, and N16961^w^ contain a disrupted *lacZ* gene. Mutation of *lacZ* was accomplished using the suicide vector pJL1. *E. coli* SM10 λpir/pJL1 was mixed at a ∼1∶1 ratio with the recipient *V. cholerae* strains and incubated on a prewarmed LB agar plate for 6 hours at 37°C. Bacterial mixtures were harvested and resuspended in 1 mL LB and subjected to 4 serial dilutions; 200 µL of diluted sample were spread onto selective LB agar plates. Transconjugants were restreaked onto LB containing ampicillin and 40 µg·mL^−1^ 5-bromo-4-chloro-3-indolyl-β-D-galactopyranoside (x-gal) to ensure disruption of *lacZ* resulted in white colony formation.

### CFU recovery time course

Strains grown overnight were back-diluted 1∶100 in selective LB broth with 0.1% arabinose. At 0, 2, 4, 6, and 8 h, a 200 µL sample was taken and serially diluted. 10 µL of each dilution was spotted onto selective LB plates and incubated overnight at 37°C.

### Membrane potential assay and flow cytometry

The BacLight Bacterial Membrane Potential Kit (Molecular Probes, Invitrogen) was used to determine whether VasX, when provided with a Sec signal peptide (SecP) to export SecP::VasX to the periplasm, caused dissipation of the membrane potential. Overnight cultures of the indicated strains were back-diluted 1∶30 in selective LB with arabinose (0.1%) and grown for 2 h at 37°C with shaking (225 rpm). Cells were diluted to ∼10^6^ cells·mL^−1^ in filtered PBS. Carbonyl cyanide *m*-chlorophenyl hydrazone (CCCP, depolarizing control) and 3,3-diethyloxacarbocyanine iodide (DiOC_2_(3)) stain were added (where appropriate) to final concentrations of 5 µM and 15 µM, respectively, and incubated for 30 min in the dark. Stained cells were analyzed using a BD LSR II flow cytometer with a 488 nm laser and PE-Texas Red (601 long pass and 616/23 band pass filters) and FITC (502 long pass and 525/50 band pass filters) detectors. Forward and side scatter and fluorescence data were collected with logarithmic signal amplification. Red/green ratios were calculated based on double positive (Texas Red and FITC) cells by collecting 50,000 events per strain tested.

### Flow cytometry analysis of cells incubated with propidium iodide

Overnight bacterial cultures were diluted 1∶100 in LB containing appropriate antibiotics in the presence of 0.1% arabinose. Cultures were grown under inducing conditions for 2 hours at 37°C with shaking. As a positive control for propidium iodide staining, dead cells were prepared by ethanol treatment: 1 mL of bacterial culture was centrifuged at 13,000 rpm for 3 minutes. The supernatant was aspirated and the pellet was resuspended in 1 mL of 70% ethanol and incubated for 20 minutes at room temperature. Cells were pelleted by centrifugation at 13,000 rpm for 3 minutes and the pellet was resuspended in 1 mL filtered PBS. ∼10^6^ cells (living or dead) were diluted into 1 mL of filtered PBS. 1.5 µL of 20 mM PI was added to each sample followed by incubation for 15 minutes in the dark.

Stained cells were analyzed using a BD LSRFortessa Cell Analyzer with a 488 nm laser, and a PE (550 long pass and 575/26 band pass filters) detector. Forward and side scatter and fluorescence were collected with logarithmic signal amplification. The percentage of cells permeable to PI was recorded by collecting 50,000 events per strain tested.

### Secretion profiles

Overnight bacterial cultures were diluted 1∶100 in LB (+/−0.1% arabinose) and grown to late-logarithmic phase. Bacterial pellet samples were resuspended in SDS protein sample buffer and boiled for 10 min. Culture supernatants were isolated, filter sterilized, and concentrated with 20% trichloroacetic acid. Supernatant proteins were resuspended in SDS protein sample buffer and boiled for 10 minutes. Samples were subjected to SDS-PAGE followed by western blotting with α-Hcp [5] and α-DnaK (loading and lysis controls, obtained from Stressgen) antibodies.

## Results

### Identification of three *V. cholerae* genes encoding T6SS-immunity proteins

**Figure 2:**
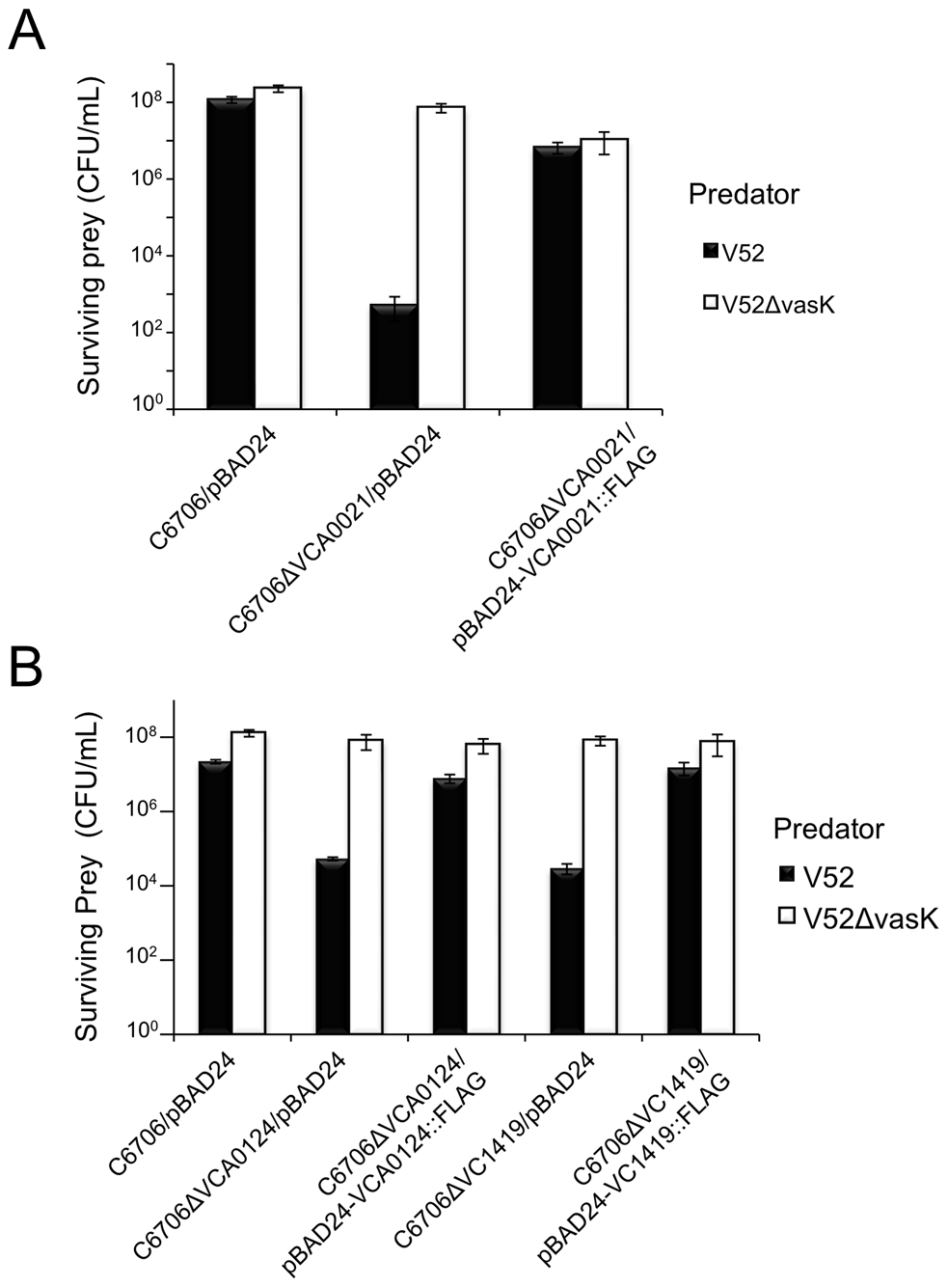
Episomal expression of VCA0021, VCA0124, and VC1419 protects respective mutants from T6SS killing. (A) Survival of rifampicin-resistant prey strains C6706 or C6706ΔVCA0021 harboring empty vector (pBAD24) or pBAD24-VCA0021::FLAG was determined by measuring CFU following exposure to the rifampicin-sensitive predator listed in the legend. V52Δ*vasK* is a T6SS-null strain that serves as a negative control for T6SS-mediated bacterial killing. Arabinose was included in all samples to drive expression from the P_BAD_ promoter. These data are representative of three independent experiments, each performed in technical duplicate. Error bars indicate the standard deviation. (B) Survival of rifampicin-resistant prey strains C6706, C6706ΔVCA0124, or C6706ΔVC1419 harboring empty vector (pBAD24), pBAD24-VCA0124::FLAG, or pBAD24-VC1419::FLAG was determined by measuring CFU following exposure to a rifampicin-sensitive predator (listed in the legend) – wild-type V52 or the T6SS-null strain V52Δ*vasK* (negative control). Arabinose was included in all samples to drive expression from the P_BAD_ promoter. These data are representative of two independent experiments, each performed in technical duplicate. Error bars indicate the standard deviation.

*V. cholerae* V52 uses its constitutively active T6SS to kill other Gram-negative bacteria such as *E. coli* strain MG1655 [16]. Interestingly, two 7th pandemic El Tor strains C6706 and N16961 (with silent T6SSs), as well as V52 are resistant to this killing phenotype. C6706 and N16961 contain a full complement of T6SS-encoding genes; however, when spotted on nutrient agar plates, these strains do not produce structural components (e.g., Hcp and VgrGs) required for the assembly of a functional T6SS. Therefore, C6706 and N16961 do not engage in T6SS-mediated killing of prokaryotes or eukaryotes under laboratory conditions [18], [34], [63]. We hypothesized that C6706 and N16961 (and other *V. cholerae* strains with silent T6SSs) encode immunity proteins within theT6SS gene clusters that are produced in a silent (T6SS-off) state. Dong *et al.* applied a high-throughput parallel sequencing technique to address this problem and identified the T6SS immunity protein-encoding genes, *tsiV1* (VC1419), *tsiV2* (VCA0021), and *tsiV3* (VCA0124), along with their corresponding T6SS effector genes *tseL* (VC1418), *vasX* (VCA0020), and *vgrG-3* (VCA0123), respectively [11]. We independently identified the same immunity genes by screening a subset of a transposon library of C6706 [58] to identify insertions within the three T6SS gene clusters that result in the loss of T6SS immunity against V52. Mutation of VCA0021 (*tsiV2*), VCA0124 (*tsiV3*), and VC1419 (*tsiV1*) [isolated as a polar mutation in VC1418 (*tseL*)] resulted in sensitivity to killing by V52 (Figure S1). We noted that mutants with transposons in VCA0114, VCA0115, and VCA0121 also became susceptible to killing by V52. However, we decided not to analyze these three genes further because deletion of these genes in strain V52 [64], which employs a constitutively active T6SS, does not interfere with the protection from killing by V52 kin bacteria. Episomal expression of VCA0021 (*tsiV2*) and VCA0124 (*tsiV3*) protects the respective C6706 mutants from T6SS-mediated killing by V52 (Figures 2A and 2B). Complementation of the VC1418 null-mutation did not restore immunity (Figure S2). However, episomal expression of VC1419 in C6706ΔVC1418 restored immunity to killing by V52 (Figure S2), implying that VC1419 is the third immunity protein-encoding gene and confirming the findings by Dong *et al.* [11]. Subsequently, in-frame deletion of VC1419 rendered C6706 susceptible to killing by V52 and this could be complemented by expression of VC1419 *in-trans* (Figure 2B).

The bacterial killing mechanism of VasX has not been characterized. Thus, we focused on the T6SS gene cluster encoding *vasX* and *tsiV2* to understand the mode-of-action of VasX and the ability of C6706 to employ TsiV2 to achieve immunity to VasX-mediated toxicity in the T6SS-off state.

### VasX is required for T6SS-mediated bacterial killing

**Figure 3:**
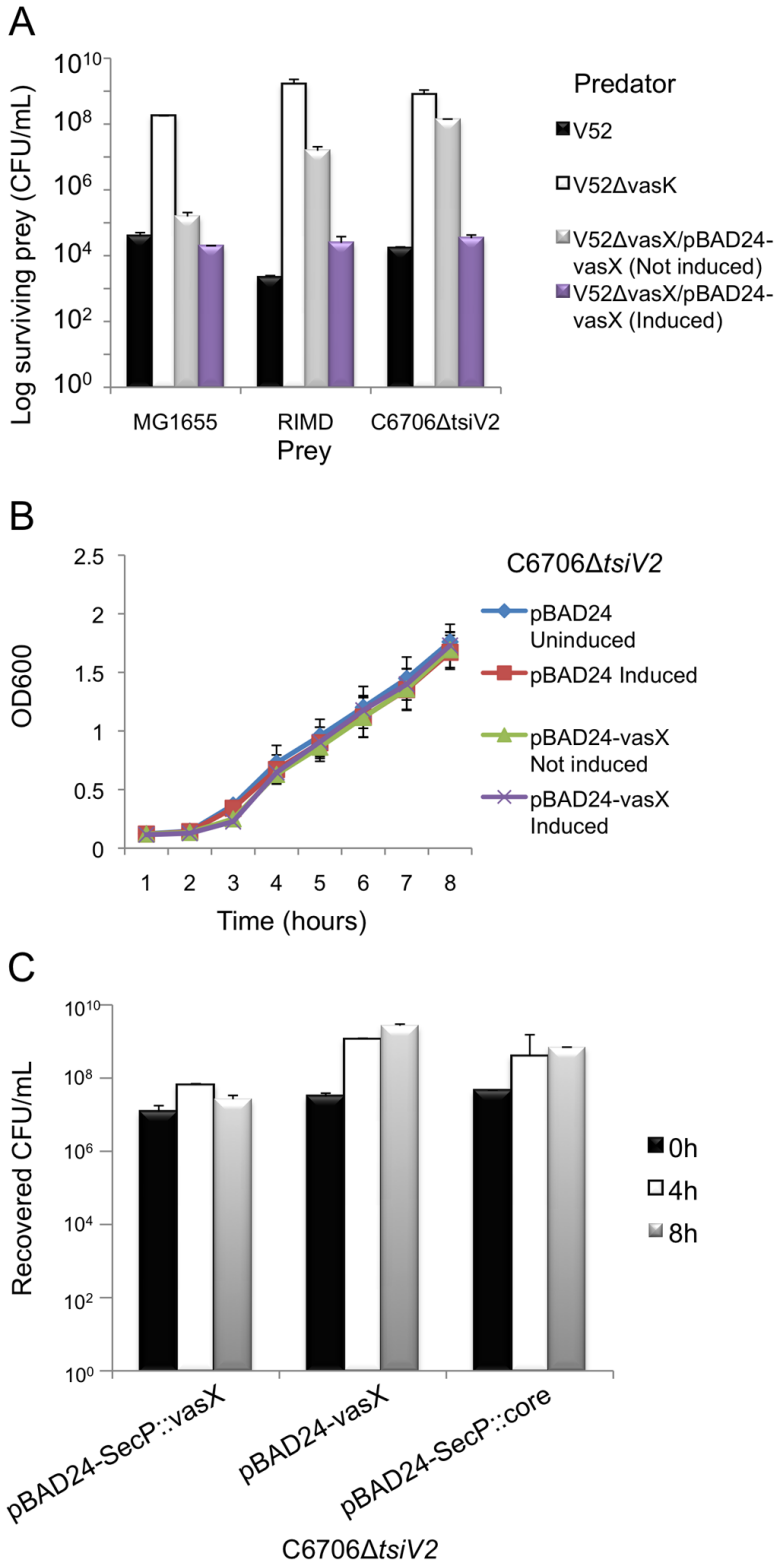
VasX is a bacterial toxin that is lethal when presented from the periplasm. (A) VasX is required for killing *V. parahaemolyticus* RIMD and C6706Δ*tsiV2*, but not *E. coli* MG1655. Survival of rifampicin-resistant prey (listed on the *x*-axis) was determined by counting CFU following exposure to the indicated rifampicin-sensitive predator (legend). V52Δ*vasK* is a T6SS-null strain and was used as a negative control. Arabinose was included where indicated (“induced”) to drive expression from the P_BAD_ promoter. These data are representative of three independent experiments, each performed in technical duplicate. Error bars indicate the standard deviation. (B) Episomal expression of *vasX* does not affect bacterial growth. C6706Δ*tsiV2* harboring the plasmids indicated in the legend were grown in liquid culture and the OD_600_ was measured at the time points indicated on the *x*-axis. Arabinose was included where indicated (“induced”) to drive expression from the P_BAD_ promoter. These data represent two independent experiments performed in technical triplicate. Error bars indicate the standard deviation. (C) Delivery of VasX to the periplasm is toxic to the producing cell. C6706Δ*tsiV2* harboring the plasmids indicated on the *x*-axis were grown for 8 hours in liquid culture. The recovered CFU/mL were enumerated after harvesting samples at the time points indicated in the legend. Arabinose was included in all samples to drive expression from the P_BAD_ promoter. These data represent three independent experiments performed in technical duplicate. Error bars indicate the standard deviation.

Secondary structure predictions indicated that VasX has homology to colicins [64]. Because we previously observed that V52 kills *E. coli* MG1655 in a T6SS-dependent manner, we first tested whether VasX was required for this killing phenotype. As shown in Figure 3A, V52 with a disabled T6SS due to a *vasK* null-mutation (V52Δ*vasK*) was unable to kill *E. coli*; however, V52 with a native *vasK* but lacking *vasX* retained the ability to kill *E. coli* to the same extent as wild-type V52. Thus, we concluded that VasX is dispensable for V52's ability to kill *E. coli*, probably because the other two T6SS effectors, VgrG-3 and TseL, compensate for the absence of VasX. To test whether VasX is important for killing *E. coli* in the absence of VgrG-3 and TseL, we performed a killing assay with a V52 predator that does not produce TseL and VgrG3, and relies on VasX for T6SS-mediated virulence. We observed that this predator strain was able to reduce the number of viable *E. coli* by ∼10-fold compared to fully attenuated V52Δ*vasK* or V52 missing all three T6SS effector genes (Figure S3). Although predator strains using VasX or VgrG-3 as the only T6SS toxin exhibited a similar Hcp secretion defect, their killing ability differed significantly (Figure S3), suggesting that VasX contributes to killing of *E. coli*. In conclusion, these data suggest that VasX is sufficient to kill *E. coli* when it is the sole effector utilized by V52, but VgrG-3 and TseL can compensate for the lack of VasX toxicity.

We determined that *V. cholerae* C6706 lacking *tsiV2*, the gene encoding the immunity protein for VasX, became susceptible to killing by V52 (Figure 3A). This killing occurred in a VasX-dependent manner such that TsiV2 conferred immunity specifically to VasX (Figure 3A). We also tested whether VasX was involved in killing of other *Vibrio* species, namely *Vibrio parahaemolyticus* strain RIMD2210633 (hereafter referred to as *V. parahaemolyticus* RIMD). *V. parahaemolyticus* RIMD encodes a functional T6SS [65], [66]; however, the ability of this strain to engage in T6SS-dependent killing of bacterial neighbors has yet to be determined. As shown in Figure 3A, VasX is required for maximal killing of *V. parahaemolyticus* RIMD as a *vasX* mutant of V52, still capable of employing the T6SS effectors TseL and VgrG-3, is attenuated with regard to the killing of *V. parahaemolyticus* RIMD. As expected, episomal expression of *vasX* complemented the killing phenotype. Other *Vibrio* species including *Vibrio alginolyticus* and *Vibrio fischeri* were also susceptible to VasX-mediated killing (data not shown). Episomal expression of the immunity protein-encoding gene *tsiV2* in *V. parahaemolyticus* RIMD provided intermediate protection against killing by V52 (Figure S4). In contrast, production of TsiV2 in MG1655 did not provide protection against VasX-mediated killing (data not shown).

Taken together, these data indicate that although VasX is not required for V52 to kill *E. coli* MG1655 due to the compensatory effects of VgrG-3 and TseL, VasX is important for killing other *Vibrio* species.

### VasX targets the inner membrane of prey bacteria

The *V. cholerae* VasX protein is important for T6SS-dependent killing of other bacteria [11] (Figure 3A) and has homology to colicins [64]. Colicins can kill target cells by degrading DNA and RNA, by forming pores in the inner membrane, or by inhibiting murein synthesis. Previous bioinformatic analysis suggested that VasX has three transmembrane domains in its C-terminus [21]; therefore, we hypothesized that the toxic activity of VasX involves damage to the inner membrane of target cells. To be toxic, pore-forming colicins must be presented to the target cell from the periplasmic face [56], [57]. Fittingly, we observed that VasX expression was not toxic towards *V. cholerae* C6706 lacking *tsiV2* (Figure 3B). To ensure that VasX was produced, we collected a lysate sample from each sample presented in Figure 3B and performed a western blot with antibodies against VasX and DnaK (loading control) (Figure S5). As we detected VasX under inducing conditions in live bacteria, we reasoned that VasX is not toxic to the producing cell, because to be toxic it has to be presented to the inner membrane of target cells from the periplasmic space similar to pore-forming colicins.

We then determined whether VasX is toxic when presented to bacteria from the periplasmic face. As shown in Figure 3C, providing VasX with a SecP secretion signal to target SecP::VasX to the periplasm reduced the growth rate of C6706 lacking *tsiV2*. The observed toxicity caused by SecP::VasX was not due to increased export of proteins to the periplasm as VgrG-3 (missing the peptidoglycan binding domain) [12] targeted to the periplasm (denoted as “SecP::core”) was not toxic to the producing cell. We concluded from this data that VasX targeted to the periplasm of the producing cell is toxic to that cell.

Knowing that periplasmic localization of VasX resulted in autotoxicity, we postulated that VasX inserts into the inner membrane of target cells similar to pore-forming colicins that share homology with VasX. To test whether production of SecP::VasX compromised membrane integrity of the producing cell, we performed an SDS lysis assay [67] and determined that production of SecP::VasX increased the sensitivity of a *tsiV2* mutant of C6706 to SDS compared to the control strains (data not shown). This finding suggests that periplasmic VasX compromises membrane integrity.

**Figure 4:**
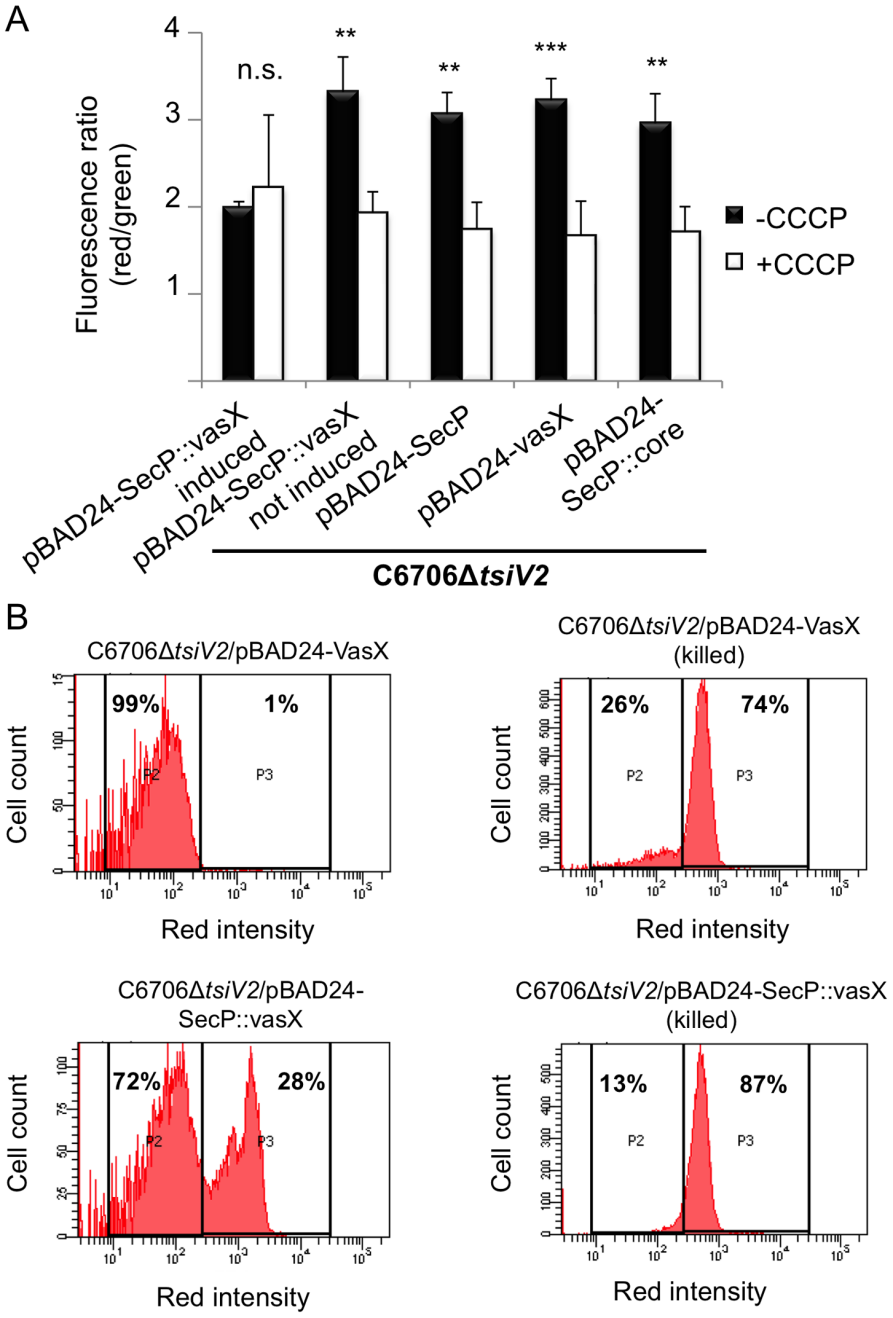
VasX compromises the integrity of the inner membrane in target cells. (A) VasX dissipates the target cell's membrane potential. C6706Δ*tsiV2* harboring the plasmids indicated on the *x*-axis were analyzed using the BacLight Membrane Potential Kit and flow cytometry. The red/green fluorescence ratio was calculated for each condition. Carbonyl cyanide *m*-chlorophenyl hydrazone (CCCP) is a chemical that uncouples the proton gradient and was used as a positive control for dissipation of membrane potential in this experiment. Arabinose was included in all samples (except in the sample noted as “not induced”) to drive expression from the P_BAD_ promoter. These data are representative of three independent experiments. Error bars indicate standard deviation. *** = p<0.001, ** = p<0.005 relative to SecP::vasX (induced, -CCCP). p-values were calculated using the Student's one-tailed, paired t-test. (B) Cells producing periplasmic VasX are permeable to propidium iodide (PI). The strain indicated at the top of each histogram (living or ethanol-killed) was incubated in the presence of PI and analyzed by flow cytometry. P2 represents cells not permeable to PI and P3 represents cells permeable to PI. The percentage of cells represented in P2 and P3 populations is indicated. These data represent three independent experiments.

If VasX resembles a pore-forming colicin, insertion into the inner membrane would result in increased cellular permeability, ion leakage, and dissipation of the bacterial membrane potential. To test whether production of SecP::VasX dissipates the membrane potential in the producing cell, we used the BacLight Bacterial Membrane Potential Kit (Molecular Probes, Invitrogen). In this experiment, the fluorescent dye DiOC_2_(3) was used to stain all cells green. If the cell is actively respiring, the dye accumulates within the cell and shifts towards red emission. Stained cells were analyzed by flow cytometry using Texas Red and FITC filters and the red/green ratio was indicative of the strength of the membrane potential in analyzed cells. CCCP, which uncouples the proton gradient, served as a positive control for dissipation of membrane potential. Production of SecP::VasX in a *tsiV2* mutant of C6706 resulted in a lower red fluorescence compared to cells producing cytoplasmic VasX, SecP-tagged VgrG-3 core (SecP::core), or empty vector control (Figure S6). When CCCP was added, the red fluorescence intensity decreased to levels comparable to cells producing SecP::VasX, implying that SecP::VasX disrupts the membrane potential to an extent similar to CCCP. Calculation of the red/green ratios indicated that the membrane potential for cells producing SecP::VasX is significantly reduced compared to controls (Figure 4A). Furthermore, SecP::VasX uncoupled the proton gradient to the same extent as the positive control CCCP (Figure 4A). Thus, only when VasX was presented from the periplasmic face, did it compromise the integrity of the inner membrane and dissipate the membrane potential (Figure 4A).

Next, we tested whether SecP::VasX made the *tsiV2* null-mutant of C6706 permeable to propidium iodide (PI) – an intercalating agent bound to a fluorescent molecule that binds DNA. Normally, PI is used to assess bacterial cell viability because it is membrane impermeant and does not penetrate healthy cells. However, in the case of dead cells, or those with damaged membranes, PI enters the cell and binds DNA. We hypothesized that cells producing SecP::VasX would be permeable to PI because they have damaged membranes. The *tsiV2*-mutant of C6706 producing SecP::VasX or VasX was grown in liquid culture in the presence of arabinose and then incubated with PI. As a positive control for PI uptake, both strains were killed by incubation in ethanol following incubation with PI. Cells were analyzed by flow cytometry to assess the permeability of these cells at a population level. Both strains that were killed in ethanol exhibited increased permeability to PI (74% of the total population for cells producing VasX and 87% of the cell population for those producing SecP::VasX) (Figure 4B). ∼30% of cells producing SecP::VasX (without ethanol incubation) showed greater uptake of PI compared to those producing VasX (Figure 4B). This indicates that production of SecP::VasX increases cellular permeability to PI. We thus conclude that VasX is a bacterial colicin-like effector that disrupts the inner membrane of target cells.

### VasW is an accessory protein of VasX

**Figure 5:**
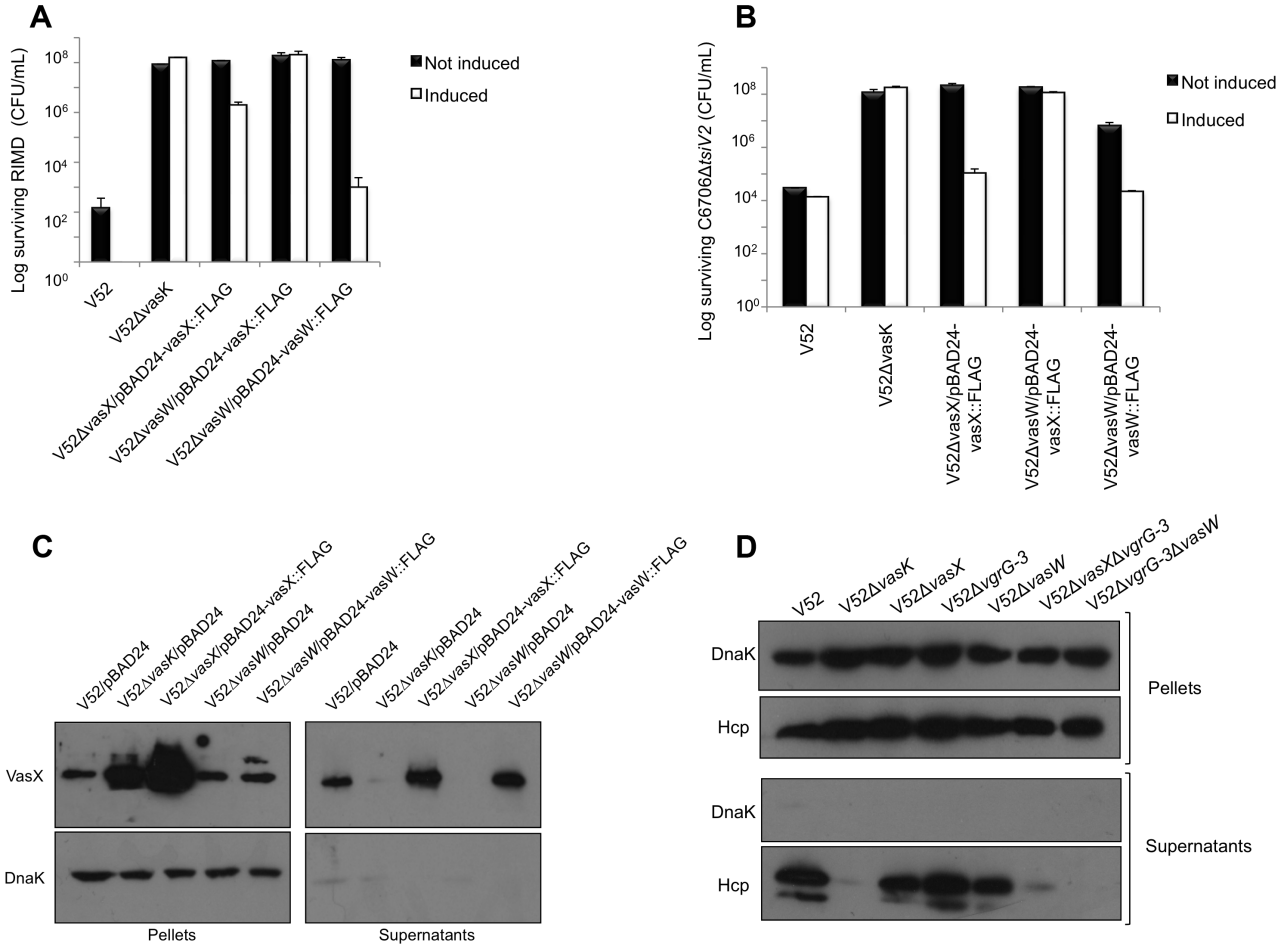
VasW plays an accessory role in VasX-mediated bacterial killing. (A) VasW is required for V52 to kill *Vibrio parahaemolyticus* RIMD. Survival of rifampicin-resistant RIMD was determined by measuring CFU following exposure to the indicated rifampicin-sensitive predator listed on the *x*-axis. Arabinose was included where indicated (“induced”) to drive expression from the P_BAD_ promoter. These data represent three independent experiments. Error bars indicate the standard deviation. (B) VasW is required for V52 to kill C6706Δ*tsiV2*. Survival of rifampicin-resistant C6706Δ*tsiV2* was determined by measuring CFU following exposure to the indicated rifampicin-sensitive predator listed on the *x*-axis. Arabinose was included where indicated (“induced”) to drive expression from the P_BAD_ promoter. These data represent three independent experiments. Error bars indicate the standard deviation. (C) V52Δ*vgrG-3* with deletions in *vasW* or *vasX* does not secrete Hcp. Bacterial pellet and supernatant samples from the strains indicated at the top of the blot were subjected to western blotting with α-Hcp and α-DnaK (loading and lysis control) antibodies. (D) V52Δ*vasW* does not secrete VasX. Bacterial pellet and supernatant samples from the strains indicated at the top of the blot were subjected to western blotting with α-VasX and α-DnaK (loading and lysis control) antibodies.

The *V. cholerae vasX* gene is contained within a satellite T6SS-cluster along with *hcp-2*, *vgrG-2*, *tsiV2*, and *vasW* (Figure 1A). Speculating that the gene products act as a cooperative unit, we set out to determine whether VasW (encoded immediately upstream of *vasX*) was important for bacterial killing. We deleted *vasW* from the V52 chromosome and tested this mutant's ability to kill *V. parahaemolyticus* RIMD. As shown in Figure 5A, deletion of *vasW* resulted in an attenuated phenotype towards *V. parahaemolyticus* RIMD, similar to the result observed when the V52 strain lacking *vasX* was used as the predator (Figure 5A). Episomal expression of *vasX* in the *vasW* null-mutant of V52 did not restore killing, demonstrating that the *vasW* mutation did not display a polar effect on *vasX*. Episomal expression of *vasW* restored killing of *V. parahaemolyticus* RIMD comparable to wild-type levels (Figure 5A). Based on these results, we hypothesized that VasW is important for VasX function.

To determine whether the lack of bacterial killing by the *vasW* null-mutant of V52 resulted from impairment of VasX function, we used the *vasW* null-mutant V52Δ*vasW* as predator in a killing assay against a C6706 mutant that lacked the immunity protein-encoding gene *tsiV2* and that had been shown to be sensitive to VasX-mediated killing by V52 [11]. As shown in Figure 5B, the killing ability of V52Δ*vasW* towards C6706Δ*tsiV2* was attenuated and this phenotype could be complemented by expression of *vasW* from the plasmid pBAD24 (Figure 5B). Therefore, we conclude that *V. cholerae* VasW assists in VasX-mediated killing of other bacteria and this defect in killing could result from improper localization of VasX or the inability of VasX to be secreted.

We previously determined that VasX depends on other T6SS proteins such as VgrG-2 and Hcp for secretion into culture supernatants [21]. Given that deletion of *vasW* results in similar phenotypes compared to V52Δ*vasX*, we asked whether VasW was required for V52 to secrete VasX. As shown in Figure 5C, the *vasW* null-mutant of V52 was unable to secrete VasX into culture supernatants, and episomal expression of *vasW* was able to restore VasX secretion. Because VasX secretion requires Hcp translocation [21], we tested whether Hcp could be found in culture supernatants of the *vasW* mutant. As expected, wild-type V52 secreted Hcp into culture supernatants, while V52 lacking *vasK* did not (Figure 5D). Hcp secretion was not affected by deletion of *vasW* in V52 (Figure 5D). We and others previously observed that V52 lacking both *vasX* and *vgrG-3* is devoid of Hcp secretion, whereas V52 lacking either *vgrG-3* or *vasX* retains the ability to secrete Hcp (Figure 5D and [11]). Because VasW appears to be important for VasX function, we tested whether a V52 double mutant lacking *vgrG-3* and *vasW* was unable to secrete Hcp, similar to what we observed for a V52 mutant lacking *vasX* and *vgrG-3*. We observed that the absence of *vgrG-3* and *vasW* abolished Hcp secretion (Figure 5D), suggesting that VasX and VasW act in a coordinated fashion. Taken together, these data indicate that VasW plays a crucial role in VasX secretion and in its function as a bacterial effector.

### *VasX* possesses an internal promoter for *tsiV2*

**Figure 6:**
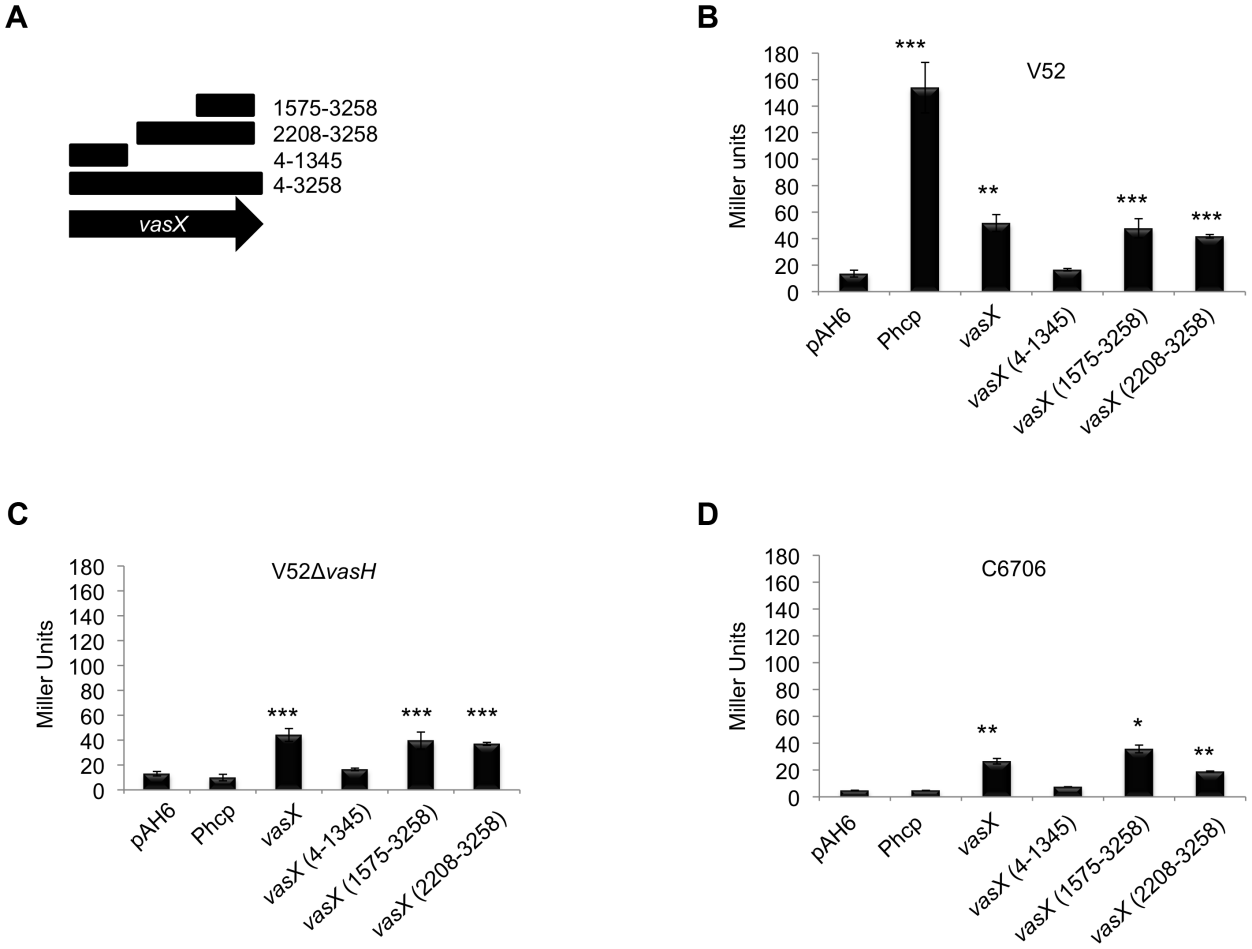
Dual expression profile of the immunity protein-encoding gene *tsiV2*. (A) Schematic representation of *vasX* fragments cloned upstream of *lacZ* in the plasmid pAH6. (B, C, D) A promoter exists within *vasX*. β-galactosidase assays were performed using the strains indicated at the top of the graph. Fragments of *vasX*, or the *hcp-2* promoter present in pAH6 are indicated on the *x*-axis. Data are representative of two independent experiments performed in triplicate and the error bars indicate the standard deviation. *** = p<0.001, ** = p<0.005, * = p<0.01 relative to the empty vector control. p-values were calculated based on the Student's one-tailed, paired t-test.

Given that C6706 does not have an active T6SS under laboratory conditions (i.e., does not produce VasX or Hcp), we hypothesized that expression of the immunity protein-encoding gene *tsiV2* does not require the T6SS regulator VasH, an alternate sigma factor σ^54^ that works at the operon promoter (located in the 400 base pairs upstream of *hcp-2*) to drive expression of *hcp-2*, *vgrG-2*, *vasX*, and likely *tsiV2* [18], [19]. This operon promoter is not active in C6706 as evidenced by a lack of Hcp production under standard laboratory conditions [34]. We speculated that a promoter exists within *vasX* capable of driving transcription of the downstream gene *tsiV2*, which would explain why wild-type C6706 cells are immune to killing by V52. To test this, we made transcriptional fusions of full-length *vasX* (4-3258, lacking the start codon) and different *vasX* truncations (nucleotides 4-1345, 1575-3258, and 2208-3258) to promoterless *lacZ* in the plasmid pAH6, as outlined in Figure 6A. The *hcp-2* promoter region (P_hcp_, basepairs −1 to −400 upstream of *hcp-2*) was also fused to *lacZ* as a control. Plasmids were transformed into V52, V52 lacking *vasH*, and C6706. The level of *lacZ* expression within cells was then determined by performing β-galactosidase assays.

V52 permits constitutive expression of T6SS-encoding genes from the promoter upstream of *hcp-2* as indicated by the significant amount of LacZ production driven by the operon promoter P_hcp_. Full length *vasX*, *vasX* (1575-3258), and *vasX* (2208-3258) drove expression of *lacZ* to levels significantly greater than the empty vector control by ∼5-fold, but less than P_hcp_ (Figure 6B). Deletion of *vasH* in V52 prohibited transcription from the *hcp-2* promoter, but not transcription from within full-length *vasX* (4-3258) and truncated *vasX* (1575-3258 and 2208-3258) (Figure 6C). When the reporter plasmids were transformed into the T6SS-silent strain C6706, we observed significant LacZ production in the presence of full-length *vasX*, *vasX* (1575-3258), and *vasX* (2208-3258), but not in the presence of P_hcp_ or *vasX* (4-1345) (Figure 6D). These data suggest the existence of a promoter within the 3′-end of *vasX* that can drive expression of *tsiV2* independently of VasH. Additional promoter dissection experiments demonstrated that the 3′-terminal 253-bp region of *vasX* (3006-3258) is sufficient to drive expression of a *lacZ* reporter (Figure S7).

**Figure 7:**
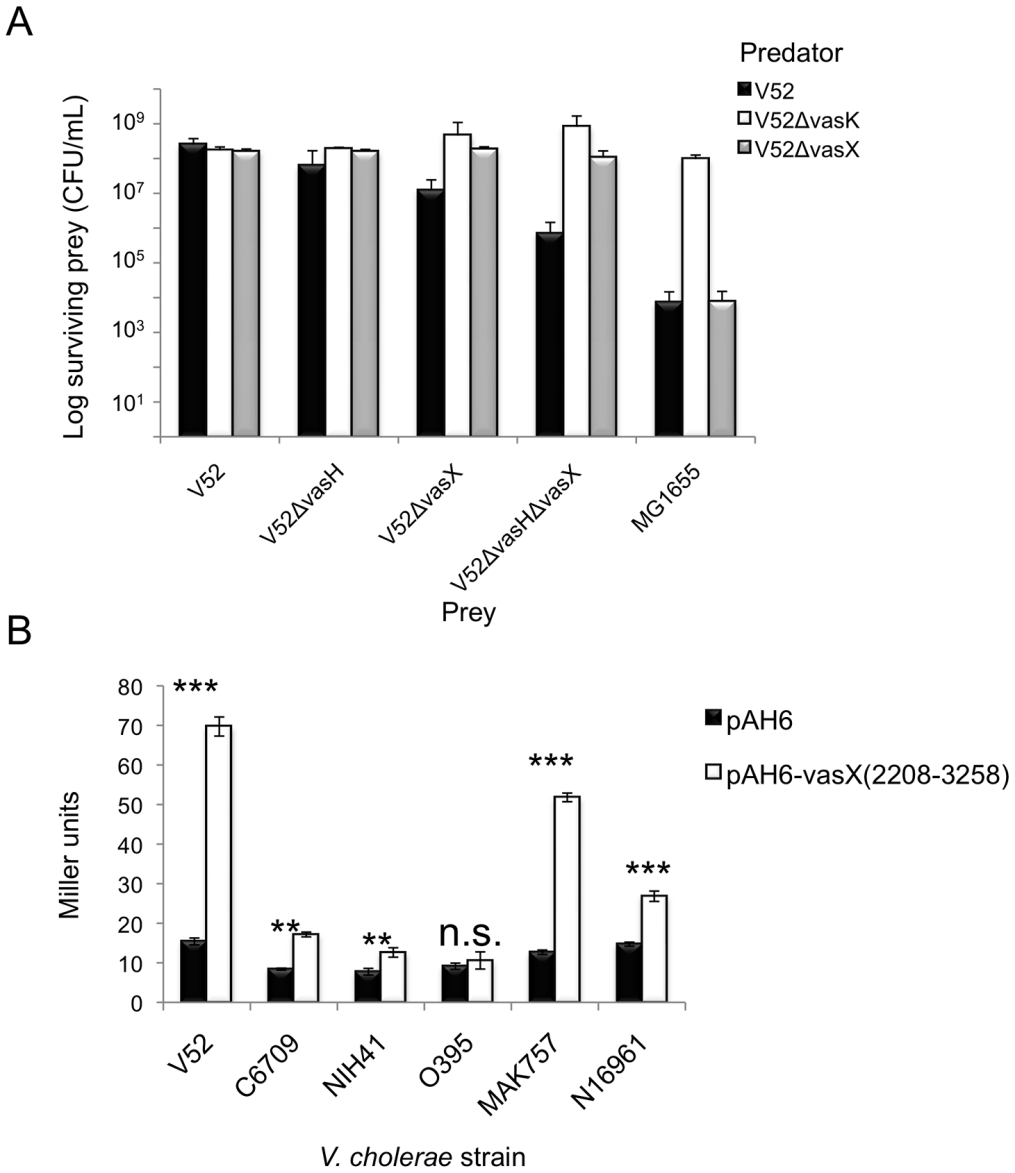
*VasX* contains a promoter to drive expression of *tsiV2* in several *V. cholerae* strain backgrounds. (A) V52 becomes susceptible to killing following deletion of *vasH* and *vasX*. Rifampicin-sensitive V52 derivatives (predator) were mixed with rifampicin-resistant V52, V52Δ*vasH*, V52Δ*vasX*, V52Δ*vasH*Δ*vasX*, and *E. coli* MG1655 (prey). Surviving prey were enumerated by selection on LB agar containing rifampicin and the results were plotted. Data are representative of three independent experiments. Error bars indicate the standard deviation. (B) The internal *tsiV2* promoter is recognized in several strains. *V. cholerae* strains indicated on the *x*-axis were transformed with pAH6-vasX(2208-3258) or plasmid control. Transformed strains were subjected to β-galactosidase assays and the Miller units were calculated and plotted. Data represent two independent experiments performed in triplicate; error bars indicate the standard deviation. *** = p<0.001, ** = p<0.005, * = p<0.01 relative to the empty vector control. n.s; not significant. p-values were calculated based on the Student's one-tailed, paired t-test.

To test the biological role of the promoter within *vasX*, we performed two independent killing assays. First, we challenged V52 lacking *vasH*, *vasX* or both with wild-type V52 and isogenic mutants lacking *vasK* or *vasX*. When challenged with a wild-type V52 predator, V52 prey and V52 prey lacking *vasH* were resistant to killing; however, V52 prey that lacked *vasX* were more susceptible to V52 predation than V52 prey that lacked *vasH* (Figure 7A). The V52 Δ*vasX*Δ*vasH* double mutant prey exhibited greater sensitivity to killing by V52 than V52 lacking either *vasX* or *vasH* (Figure 7A). Furthermore, a V52 predator lacking *vasX* was able to kill *E. coli* MG1655 but failed to kill V52 prey regardless of their respective mutations, suggesting that null mutations of *vasX* and *vasH* impaired immunity to VasX.

In the second series of killing assays, we constructed a C6706 derivative containing an in-frame deletion mutation of *vasX*, and used this strain as prey in a killing assay with a V52 predator. We reasoned that in this strain, deletion of the putative *tsiV2* promoter would render C6706 lacking *vasX* susceptible to killing by V52. C6706Δ*vasX* (with empty vector pBAD24) were killed by V52 (Figure S8). Importantly, *trans*-expression of *vasX* was unable to restore immunity to killing by V52 because expression from pBAD24 does not restore promoter function to drive expression of *tsiV2* on the C6706 chromosome (Figure S8). Expression of *tsiV2* from pBAD24 protected C6706Δ*vasX* from being killed by V52 (Figure S8). Taken together, these data indicate that the promoter within *vasX* can drive transcription of *tsiV2* in a VasH-independent manner.

To determine whether the promoter in *vasX* was functional specifically in V52 or in a variety of strains, we also transformed a pAH6 empty vector or pAH6-vasX (2208-3258) into *V. cholerae* O1 serogroup strains O395^w^, C6709^w^, NIH41^w^, MAK 757^w^, and N16961^w^. As shown in Figure 7B, V52, C6709^w^, NIH41^w^, MAK 757^w^, and N16961^w^ had significantly higher LacZ activities compared to their empty vector controls. In contrast, O395^w^ did not have higher levels of LacZ with pAH6-vasX (2208-3258) compared to the empty vector control (Figure 7B). These data indicate that the promoter found within *vasX* can elicit transcription of downstream genes in a variety of *V. cholerae* strains and that this phenomenon is not specific to V52.

### *TseL* and *vgrG-3* possess internal promoters for *tsiV1* and *tsiV3*, respectively

Based on the data presented in Figures 6 and 7, we tested whether *tseL* and *vgrG-3* also contain internal promoters to drive VasH-independent transcription of *tsiV1* and *tsiV3*, respectively. We made transcriptional fusions of *tseL* and *vgrG-3* to promoterless *lacZ* in pAH6 and transformed the plasmids into V52, V52 lacking *vasH*, and C6706. Similar to what we observed with *vasX*, both *tseL* and *vgrG-3* resulted in statistically significant production of β-galactosidase compared to the empty vector control. Production of β-galactosidase was independent of VasH (Figure S9), and the amount of β-galactosidase activity measured was higher for *vgrG-3* than for either *vasX* or *tseL* (Figures 6 and S9).

Last, we performed killing assays using C6706 lacking *tseL* or *vgrG-3* as prey to determine if deletion of the effector gene, and thus the putative promoter for the downstream immunity protein-encoding genes, rendered these strains susceptible to killing by V52. As shown in Figure S2, C6706 lacking *tseL* or *vgrG-3* (with empty vector pBAD24) were killed by V52. Episomal expression of *tseL* and *vgrG-3* in their respective mutants did not restore immunity (Figure S2). However, immunity to V52 could be restored by complementing *tseL* and vgrG-3 null-mutations in C6706 with *tsiV1* and *tsiV3*, respectively (Figure S2). Taken together, these data indicate that internal promoters located within *tseL* and *vgrG-3* can drive transcription of *tsiV1* and *tsiV3* in a VasH-independent manner.

## Discussion

TA systems are commonly employed by bacteria to ward off bacterial competitors [23], [33], [35], [37], [68]–[71]. In some bacteria, the presence of an antitoxin or immunity protein prevents self-intoxication and killing by toxins produced by sister cells. Similarly, it appears that T6SS immunity proteins protect against an oncoming attack by neighboring T6SS-active bacteria. By screening a C6706 T6SS transposon library for susceptibility to killing by *V. cholerae* V52 (Figure S1), we identified three T6SS immunity protein-encoding genes, namely *tsiV1*, *tsiV2*, and *tsiV3* (Figure 1A). These data agree with recent findings of Dong *et al.* who also identified *tsiV1*, *tsiV2*, and *tsiV3* as *V. cholerae* T6SS immunity protein-encoding genes using a Tn-seq approach [11].

Episomal expression of *tsiV2* in the *Vibrio* species *V. parahaemolyticus* RIMD provided significant protection against killing by V52 (Figure S4). In contrast, episomal expression of *tsiV2* in *E. coli* MG1655 did not provide protection against T6SS-mediated killing – even when VasX was the only bacterial effector employed (data not shown). Importantly, *V. parahaemolyticus* RIMD has a functional T6SS but does not possess homologs to VasX or TsiV2 according to BLASTn and BLASTp analyses (data not shown). MG1655 also does not possess genes similar to *vasX* and *tsiV2* (data not shown). Therefore, we postulate that even though *V. parahaemolyticus* RIMD contains T6SS gene clusters, putative immunity proteins are not cross-protective against V52 effectors. Furthermore, we suggest that TsiV2 provides (intermediate) protection in *V. parahaemolyticus* RIMD, but not in *E. coli* MG1655, because TsiV2 requires other T6SS proteins or cofactors for proper function and/or localization within the prey cell that are produced by *V. parahaemolyticus* but not *E. coli*.

In all TA systems, including those in which the toxin is a colicin, the presence of an immunity protein prevents toxicity. Commonly, the gene encoding the immunity protein is located directly adjacent to the effector gene [56], [72], [73]. Using β-galactosidase assays, we demonstrated that a promoter exists within the 1,051 nucleotides of the 3′end of *vasX* to drive expression of *lacZ* in V52, V52Δ*vasH*, C6706, C6709^w^, NIH41^w^, MAK 757^w^, and N16961^w^ (Figures 6 and 7B). The promoter was determined to be within the 3′-terminal 253-bp region of *vasX* in a subsequent experiment with strain C6706 (Figure S7). Expression driven by promoters within *vasX*, *vgrG-3*, and *tseL* in the strains tested implies that C6706 produces immunity proteins under laboratory conditions to mediate protection against a T6SS-onslaught by bacterial neighbors (Figures 6, 7, S2, S8, and S9). We are currently conducting experiments to identify the exact sequence that encodes these promoters.

Constitutive expression of T6SS immunity protein-encoding genes could be advantageous for several reasons. The most obvious reason for maintaining basal expression of genes encoding immunity proteins is for protection against an attack by sister cells. Bacteria with activated T6SS gene clusters could readily kill sister cells that live within close quarters (i.e., biofilms) and lack expression of immunity protein-encoding genes. Constitutive expression of immunity protein-encoding genes, independent of other genes encoding T6SS components, would also allow the bacterium to conserve energy as the T6SS secretion apparatus is a large, dynamic structure [7], [8], [33], the production of which likely requires vast energy expenditure. Previously it was shown that infections of infant mice with *V. cholerae* V52 unable to shut down its T6SS caused inflammation and actin cross-linking in the mouse gut [74]. By employing strict regulatory mechanisms for T6SS proteins while maintaining continuous expression of immunity protein-encoding genes (such is the case with C6706), *V. cholerae* could prevent identification by the host immune system and avoid a loss of viability at the time of T6SS activation [75].

Outside the human host, the T6SS-off state can be advantageous in mixed bacterial populations. Immunity protein-encoding genes only protect against T6SS effectors of the same species, sensitizing *V. cholerae* to T6SS effectors of other organisms including *P. aeruginosa* [33]. In a mixed population of *V. cholerae* and *P. aeruginosa*, *P. aeruginosa* employed its T6SS to kill *V. cholerae* only if *V. cholerae* used its T6SS to attack first. *V. cholerae* with an inactive T6SS did not induce a counter-attack from neighboring *P. aeruginosa* and the two bacterial species maintained a peaceful coexistence [33]. We speculate that maintaining constitutive expression of genes encoding immunity proteins independently from other T6SS-encoding genes provides the bacterium with a fitness advantage in the event it engages in T6SS dueling with another T6SS-active kin bacterium.

The three immunity protein-encoding genes *tsiV1*, *tsiV2*, and *tsiV3* are located directly downstream of their corresponding effector genes *tseL*, *vasX*, and *vgrG-3*, respectively. We previously demonstrated that VgrG-3 degrades the peptidoglycan of target cells [12], a phenotype that has also been described for T6SS bacterial effectors produced by *P. aeruginosa* [35]. TseL is a predicted class III lipase [11], however the role of TseL within the context of the *V. cholerae* T6SS remains to be determined. Here we showed that VasX is an effector that targets prokaryotes lacking the VasX immunity protein-encoding gene *tsiV2* and that the accessory protein VasW is required for secretion of VasX.

We previously demonstrated that *V. cholerae* V52 uses its T6SS to kill *E. coli* [16], and data presented here indicate that all three effectors – TseL, VgrG-3, and VasX are active against *E. coli*; however, the presence of TseL and VgrG-3 can compensate for the lack of VasX (Figures 3A and S3). A similar conclusion was reached by Dong *et al.* such that VasX is sufficient but not required for V52 to kill *E. coli* [11]. Interestingly, when other *Vibrio* species such as *V. parahaemolyticus* RIMD or a mutant of *V. cholerae* strain C6706 lacking *tsiV2* were used as prey, VasX had a much stronger phenotype (Figure 3A). In this case, VasX was a more effective effector against *V. parahaemolyticus* RIMD and C6706, but less effective for killing *E. coli*. This could be due to factors such as a requirement for specific receptors on target cells or varying degrees of susceptibility to T6SS effectors.

We determined that VasW, a protein whose gene lies directly upstream of *vasX*, is required for secretion of VasX and that V52 lacking *vasW* has a phenotype similar to V52 lacking *vasX*. We and others [11] demonstrated that a double mutant lacking *vasX* and *vgrG-3* fails to secrete the T6SS substrate Hcp into culture supernatants. Dong *et al.* suggested that VgrG-3 and VasX interact to form part of the T6SS secretion apparatus [11]. Because V52 lacking both *vgrG-3* and *vasW* also does not secrete Hcp (Figure 5D), we propose that VasW acts as an accessory protein responsible for mediating the interaction between VasX and structural proteins of the secretion apparatus. In light of the recently proposed mechanism for T6SS effector translocation [14], VasW could bind to the PAAR protein located at tip of the injectosome to prepare VasX for T6SS-mediated translocation. VasW-mediated recruitment of VasX to the T6SS injectosome could then function as a checkpoint for ejection of the inner Hcp tube decorated with a VgrG-3-containing tip.

Previously, VasX was reported to have homology to colicins [64], secreted proteins produced by and toxic to certain strains of *E. coli* [37]. The data presented here suggest that similar to pore-forming colicins, VasX perturbs the inner membrane of target cells. Although we cannot conclude that VasX acts via pore-formation, our data indicate that VasX dissipates the target cell's membrane potential and increases cellular permeability – both characteristics of pore-forming toxins [70], [76]–[78].

The observation that periplasmic localization of VasX is toxic to the producer cell also indicates similarities between VasX and pore-forming colicins. Cytoplasmic production of pore-forming colicins does not result in toxicity, because pore formation can occur only when the colicin is presented to the cell from the periplasmic face [56], [57], [67]. We also observed that production of VasX in C6706 that lacks the immunity protein-encoding gene *tsiV2* was not toxic to the producing cell (Figure 3B), but providing VasX with the Sec signal peptide resulted in autotoxicity (Figure 3C). Along these lines, we previously demonstrated that VasX is a secreted protein that is present in the cytoplasm and membrane fractions of V52; however, VasX is undetectable in the periplasmic fraction by western blot analysis [21]. It appears that VasX either bypasses the periplasm, or is present transiently in small quantities *en route* out of the cell, as its presence in the periplasm could be toxic.

Interestingly, cells producing SecP::VasX initially increased in cell number, but then began to die. After 8 hours of induction the bacterial concentration was similar to the starting bacterial concentration (Figure 3C). It is unclear why we did not observe a more significant reduction in the number of surviving bacteria; however, a similar growth/toxicity phenotype referred to as “quasilysis” has been described for colicin lysis proteins that perforate colicin-producing cells and release colicin molecules into the extracellular milieu [56], [79]–[82]. We propose a model whereby VasX is injected into the periplasm of target cells along with T6SS structural proteins and other putative effectors. Upon arrival in the periplasm, VasX inserts into the inner membrane permeabilizing the target cell – an activity similar to quasilysis

We previously determined that VasX has a role in T6SS-mediated virulence towards the amoeba *D. discoideum* and that VasX binds eukaryotic lipids [19]. The fact that VasX has the ability to target both prokaryotic and eukaryotic cells suggests that VasX targets a cellular structure common to both cell types such as the cytoplasmic membrane. A similar model has been proposed by the Mougous group regarding T6SS lipase effectors that target cytoplasmic membranes of both prokaryotes and eukaryotes [79]. In the case of *D. discoideum*, we propose that perturbation of the plasma membrane is responsible for the VasX-mediated phenotype observed in this eukaryotic host. Targeting the membrane rather than a single protein/receptor is an evolutionarily advantageous mechanism employed by bacterial effectors because developing resistance to this toxic mechanism would prove challenging for target cells.

## Supporting Information

Figure S1 Identification of T6SS immunity protein-encoding genes in *V. cholerae*. Killing assays were performed to screen a C6706 T6SS transposon library for mutants that became sensitive to killing by *V. cholerae* V52. Predator strains included wild-type V52 and the T6SS-null strain V52Δ*vasK* (negative control). *E. coli* strain MG1655 was included as a positive control as this strain had previously been shown to be susceptible to killing by V52. The data are presented in three individual graphs, each representing one of the *V. cholerae* T6SS gene clusters (i.e., VCA0017-VCA0021 – top panel, VCA0105-VCA0124 – middle panel, and VC1415-VC1420 – bottom panel). The competitive index was calculated by dividing recovered CFU after exposure to V52 by recovered CFU exposed to V52Δ*vasK*. Arrows indicate C6706 mutants identified as sensitive to killing by V52. The V52 strain used in this experiment does not lack *hlyA*, *hapA*, or *rtxA*. These data are representative of three independent experiments performed in technical duplicate. Error bars indicate the standard deviation.
(TIF)

Figure S2 Complementation of VC1419 (*tsiV1*) and VCA0124 (*tsiV3*) null-mutations restores immunity to killing by V52. Deletion of VCA0123 (*vgrG-3*) and VC1418 (*tseL*) renders C6706 sensitive to killing by V52. Survival of rifampicin-resistant (A) C6706ΔVC1418 and (B) C6706ΔVCA0123 harboring either empty vector (pBAD24), pBAD24-VCA0123::FLAG, pBAD24-VCA0124::FLAG, pBAD24-VC1418::FLAG, or pBAD24-VC1419::FLAG was determined by measuring CFU following exposure to the indicated rifampicin-sensitive predator (listed in the legend). These data are representative of two independent experiments performed in technical duplicate. Error bars indicate the standard deviation.
(TIF)

Figure S3 VasX is sufficient but not required for killing of *E. coli*. (A) Survival of rifampicin-resistant *E. coli* was determined by enumerating CFU following exposure to the indicated rifampicin-sensitive predator (listed on the *x*-axis). V52 and V52 derivatives used in this experiment do not lack *hlyA*, *hapA*, or *rtxA*. These data are representative of two independent experiments performed in technical duplicate. Error bars indicate the standard deviation. (B) V52Δ*vgrG-3*Δ*tseL* secretes Hcp. Pellet and supernatant samples were prepared using mid-logarithmic cultures of the strains indicated at the top of the blot. Samples were subjected to SDS-PAGE followed by western blotting with Hcp and DnaK (loading and lysis control) antibodies. Molecular weight is noted to the left of the blot. V52 and V52 derivatives in this experiment do not lack *hlyA*, *hapA*, or *rtxA*. These data are representative of three independent experiments.
(TIF)

Figure S4 *Trans*-expression of *tsiV2* in *Vibrio parahaemolyticus* RIMD results in partial protection from killing by V52. Survival of rifampicin-resistant RIMD harboring either empty vector or pBAD33-tsiV2::6×His was determined by measuring CFU following exposure to the indicated rifampicin-sensitive predator (listed in the legend) in the presence of arabinose (to drive expression from the P_BAD_ promoter). These data are representative of two independent experiments. Error bars indicate the standard deviation. ** = p<0.005 relative to empty vector control (vs. V52). p-values were calculated using the Student's one-tailed, paired t-test.
(TIF)

Figure S5 Western blot demonstrating VasX production from pBAD24-vasX in C6706Δ*tsiV2*. Pellet samples of C6706Δ*tsiV2* containing empty vector (pBAD24) or pBAD24-vasX were harvested and subjected to western blotting using VasX and DnaK (loading control) antibodies. Arabinose was included where indicated to drive expression from the P_BAD_ promoter. These data are representative of two independent experiments.
(TIF)

Figure S6 VasX dissipates the target cell's membrane potential. C6706Δ*tsiV2* expressing the genes from pBAD24 (indicated at the top of the histogram) were analyzed using the BacLight Membrane Potential Kit and flow cytometry. Carbonyl cyanide *m*-chlorophenyl hydrazone (CCCP) is a chemical that uncouples the proton gradient and was used as a positive control for dissipation of membrane potential in this experiment. Arabinose was included in all samples (except the sample noted as “not induced”) to drive expression from the P_BAD_ promoter. These data are representative of three independent experiments.
(TIF)

Figure S7 The 3′terminal 253 bp-region of *vasX* supports promoter activity. β-galactosidase assays were performed using C6706 transformed with the plasmids indicated on the *x*-axis. Miller units were calculated and plotted. These data are representative of two independent experiments performed in technical triplicate. Error bars indicate the standard deviation. *** = p<0.001, * = p<0.01 relative to the empty vector control. p-values were calculated based on the Student's one-tailed, paired t-test.
(TIF)

Figure S8 Deletion of *vasX* renders C6706 sensitive to killing by V52. Survival of rifampicin-resistant C6706Δ*vasX* and complemented strains (indicated on the *x*-axis) was determined by measuring CFU following exposure to the indicated rifampicin-sensitive predator listed in the legend. These data are representative of three independent experiments performed in technical duplicate. Error bars indicate the standard deviation.
(TIF)

Figure S9 Dual expression profile of *tsiV1* and *tsiV3*. β-galactosidase assays were performed using the strains indicated at the top of each graph that had been transformed with the plasmids indicated on the *x*-axis. Miller units were calculated and plotted. These data are representative of two independent experiments performed in technical triplicate. Error bars indicate the standard deviation. *** = p<0.001, ** = p<0.005 relative to the empty vector control. p-values were calculated based on the Student's one-tailed, paired t-test.
(TIF)
