# Supplementary figures and images for: Dual Expression Profile of Type VI Secretion System Immunity Genes Protects Pandemic Vibrio cholerae
Source: PLoS Pathog. 2013 Dec 5;9(12):e1003752. doi: 10.1371/journal.ppat.1003752 (PMC3857813; doi:10.1371/journal.ppat.1003752)

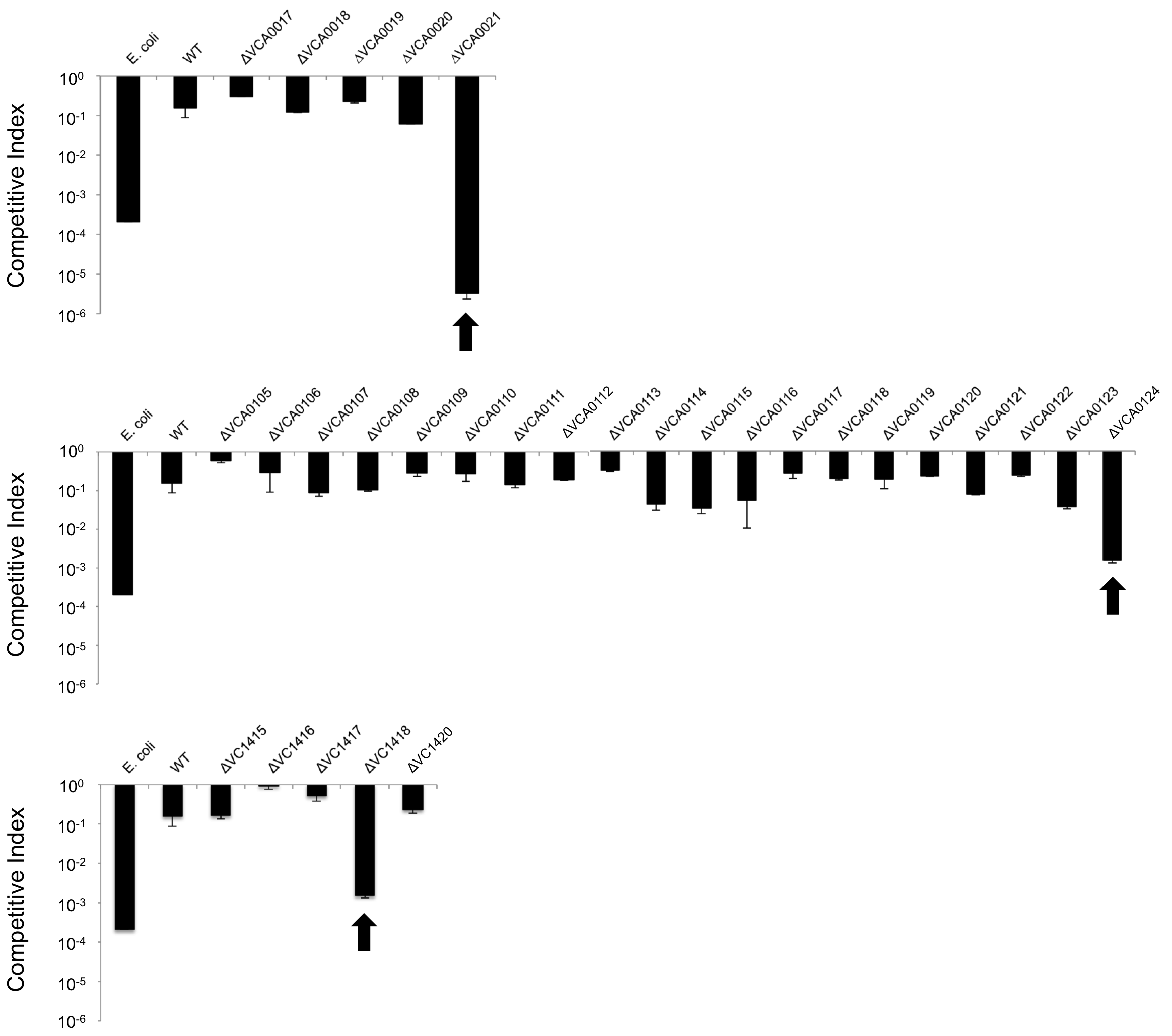

Supplement: Figure S1 — Identification of T6SS immunity protein-encoding genes in V. cholerae. Killing assays were performed to screen a C6706 T6SS transposon library for mutants that became sensitive to killing by V. cholerae V52. Predator strains included wild-type V52 and the T6SS-null strain V52ΔvasK (negative control). E. coli strain MG1655 was included as a positive control as this strain had previously been shown to be susceptible to killing by V52. The data are presented in three individual graphs, each representing one of the V. cholerae T6SS gene clusters (i.e., VCA0017-VCA0021 – top panel, VCA0105-VCA0124 – middle panel, and VC1415-VC1420 – bottom panel). The competitive index was calculated by dividing recovered CFU after exposure to V52 by recovered CFU exposed to V52ΔvasK. Arrows indicate C6706 mutants identified as sensitive to killing by V52. The V52 strain used in this experiment does not lack hlyA, hapA, or rtxA. These data are representative of three independent experiments performed in technical duplicate. Error bars indicate the standard deviation. (TIF) [file ppat.1003752.s001.tif]

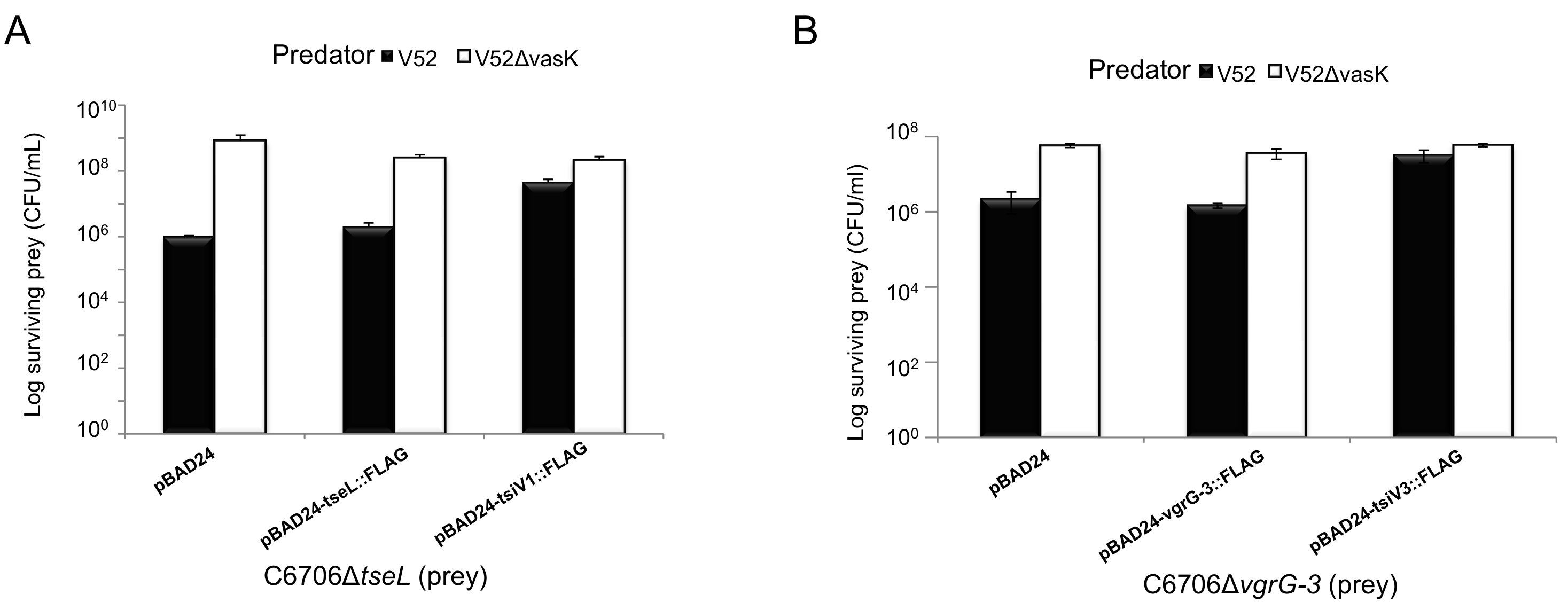

Supplement: Figure S2 — Complementation of VC1419 (tsiV1) and VCA0124 (tsiV3) null-mutations restores immunity to killing by V52. Deletion of VCA0123 (vgrG-3) and VC1418 (tseL) renders C6706 sensitive to killing by V52. Survival of rifampicin-resistant (A) C6706ΔVC1418 and (B) C6706ΔVCA0123 harboring either empty vector (pBAD24), pBAD24-VCA0123::FLAG, pBAD24-VCA0124::FLAG, pBAD24-VC1418::FLAG, or pBAD24-VC1419::FLAG was determined by measuring CFU following exposure to the indicated rifampicin-sensitive predator (listed in the legend). These data are representative of two independent experiments performed in technical duplicate. Error bars indicate the standard deviation. (TIF) [file ppat.1003752.s002.tif]

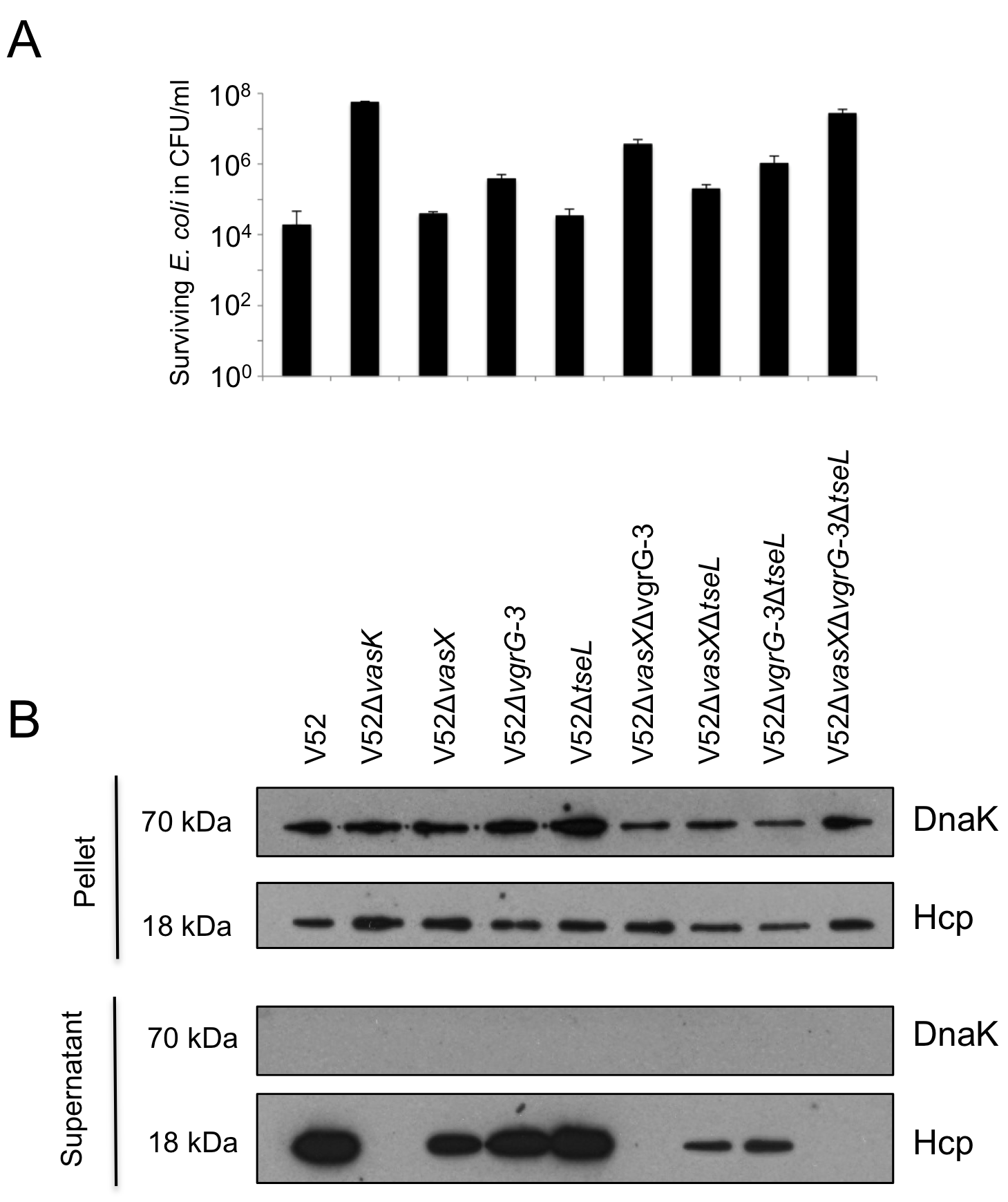

Supplement: Figure S3 — VasX is sufficient but not required for killing of E. coli. (A) Survival of rifampicin-resistant E. coli was determined by enumerating CFU following exposure to the indicated rifampicin-sensitive predator (listed on the x-axis). V52 and V52 derivatives used in this experiment do not lack hlyA, hapA, or rtxA. These data are representative of two independent experiments performed in technical duplicate. Error bars indicate the standard deviation. (B) V52ΔvgrG-3ΔtseL secretes Hcp. Pellet and supernatant samples were prepared using mid-logarithmic cultures of the strains indicated at the top of the blot. Samples were subjected to SDS-PAGE followed by western blotting with Hcp and DnaK (loading and lysis control) antibodies. Molecular weight is noted to the left of the blot. V52 and V52 derivatives in this experiment do not lack hlyA, hapA, or rtxA. These data are representative of three independent experiments. (TIF) [file ppat.1003752.s003.tif]

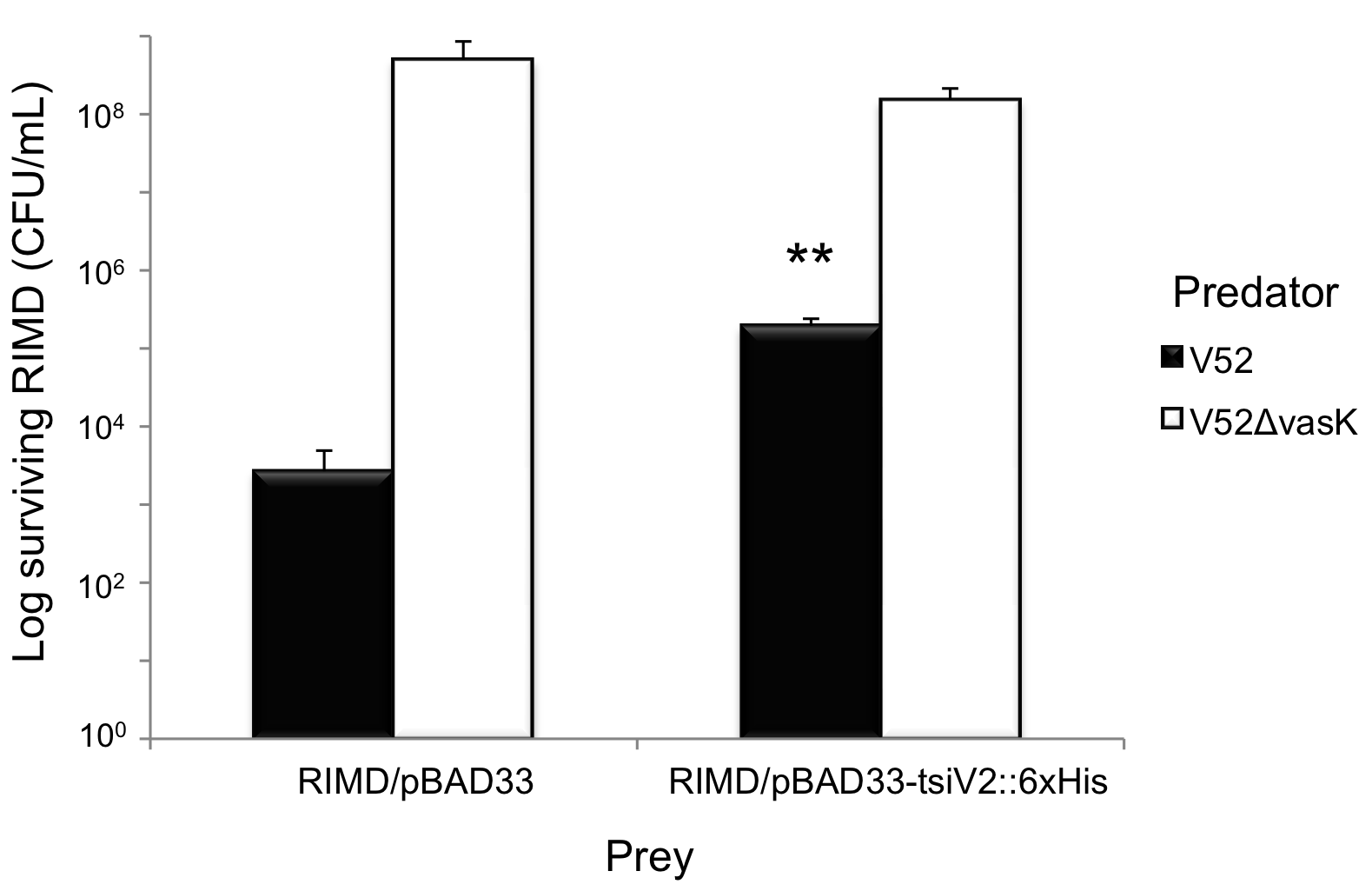

Supplement: Figure S4 — Trans-expression of tsiV2 in Vibrio parahaemolyticus RIMD results in partial protection from killing by V52. Survival of rifampicin-resistant RIMD harboring either empty vector or pBAD33-tsiV2::6×His was determined by measuring CFU following exposure to the indicated rifampicin-sensitive predator (listed in the legend) in the presence of arabinose (to drive expression from the PBAD promoter). These data are representative of two independent experiments. Error bars indicate the standard deviation. ** = p<0.005 relative to empty vector control (vs. V52). p-values were calculated using the Student's one-tailed, paired t-test. (TIF) [file ppat.1003752.s004.tif]

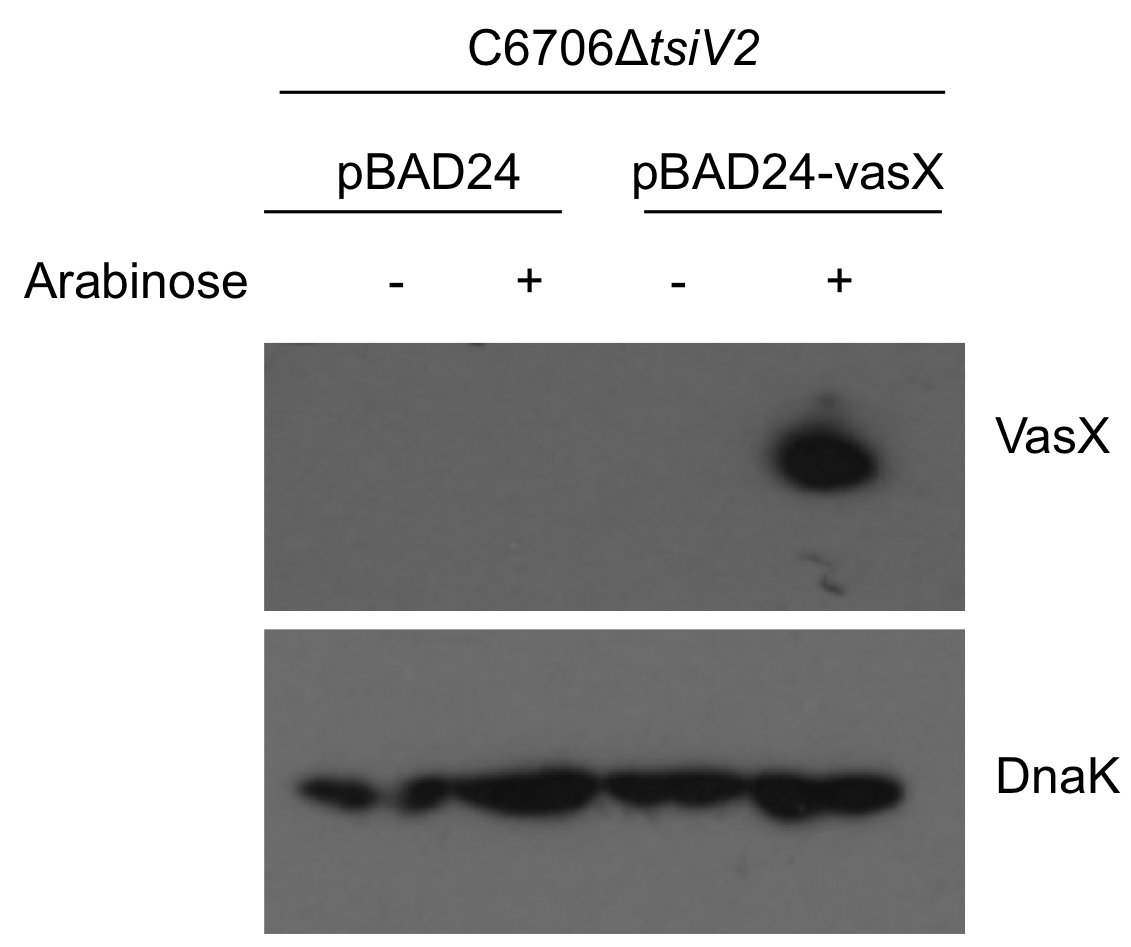

Supplement: Figure S5 — Western blot demonstrating VasX production from pBAD24-vasX in C6706ΔtsiV2. Pellet samples of C6706ΔtsiV2 containing empty vector (pBAD24) or pBAD24-vasX were harvested and subjected to western blotting using VasX and DnaK (loading control) antibodies. Arabinose was included where indicated to drive expression from the PBAD promoter. These data are representative of two independent experiments. (TIF) [file ppat.1003752.s005.tif]

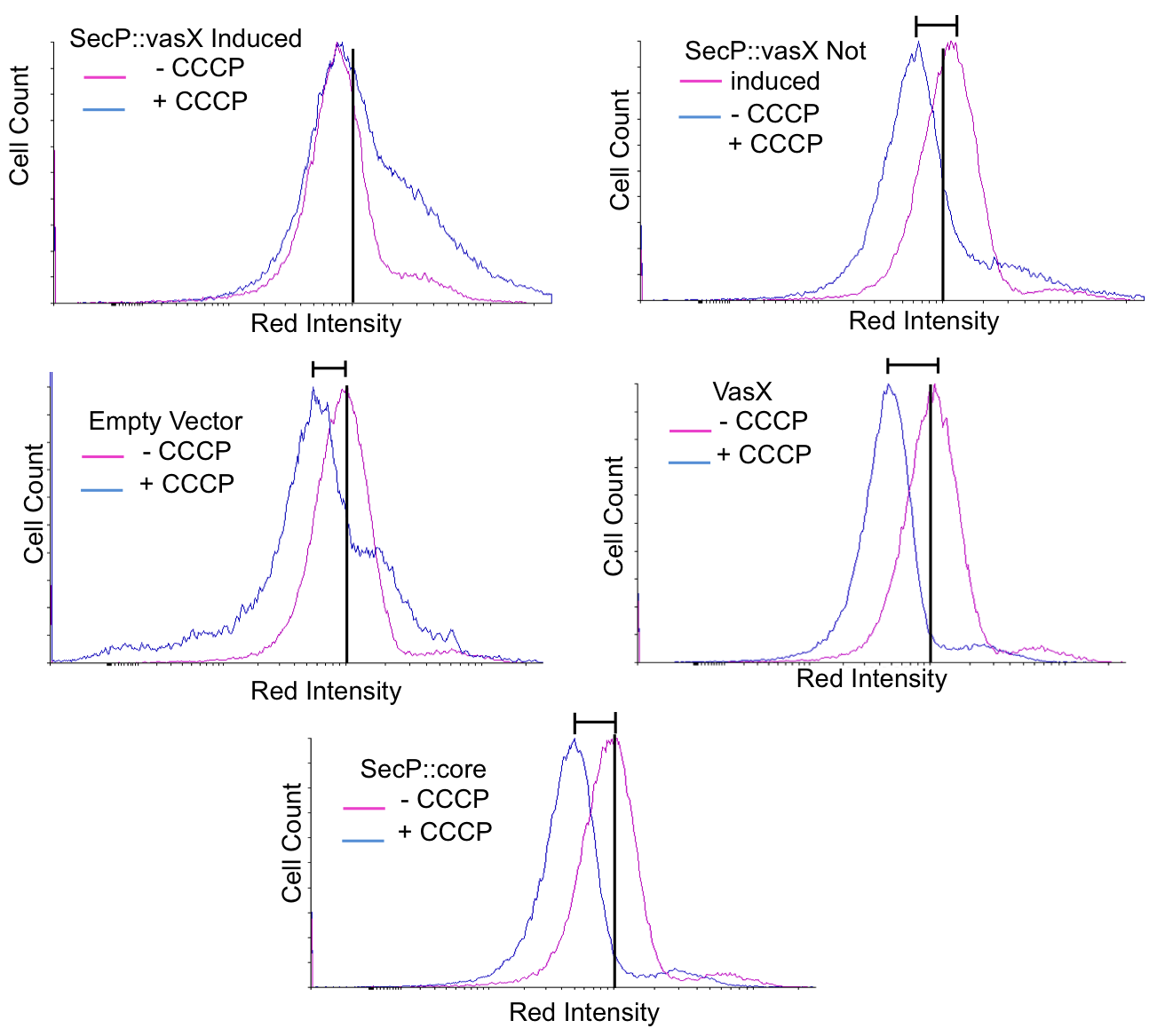

Supplement: Figure S6 — VasX dissipates the target cell's membrane potential. C6706ΔtsiV2 expressing the genes from pBAD24 (indicated at the top of the histogram) were analyzed using the BacLight Membrane Potential Kit and flow cytometry. Carbonyl cyanide m-chlorophenyl hydrazone (CCCP) is a chemical that uncouples the proton gradient and was used as a positive control for dissipation of membrane potential in this experiment. Arabinose was included in all samples (except the sample noted as “not induced”) to drive expression from the PBAD promoter. These data are representative of three independent experiments. (TIF) [file ppat.1003752.s006.tif]

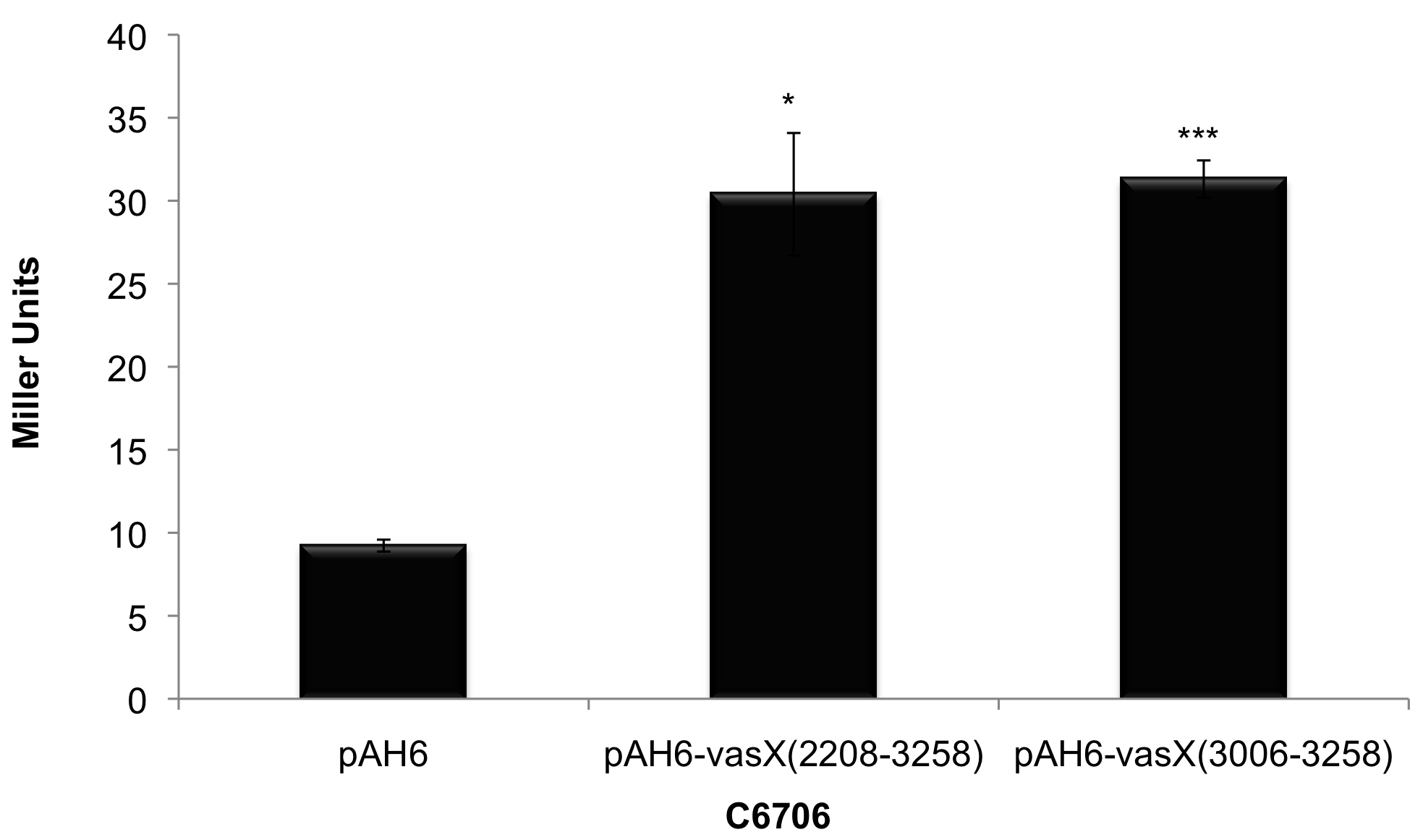

Supplement: Figure S7 — The 3′terminal 253 bp-region of vasX supports promoter activity. β-galactosidase assays were performed using C6706 transformed with the plasmids indicated on the x-axis. Miller units were calculated and plotted. These data are representative of two independent experiments performed in technical triplicate. Error bars indicate the standard deviation. *** = p<0.001, * = p<0.01 relative to the empty vector control. p-values were calculated based on the Student's one-tailed, paired t-test. (TIF) [file ppat.1003752.s007.tif]

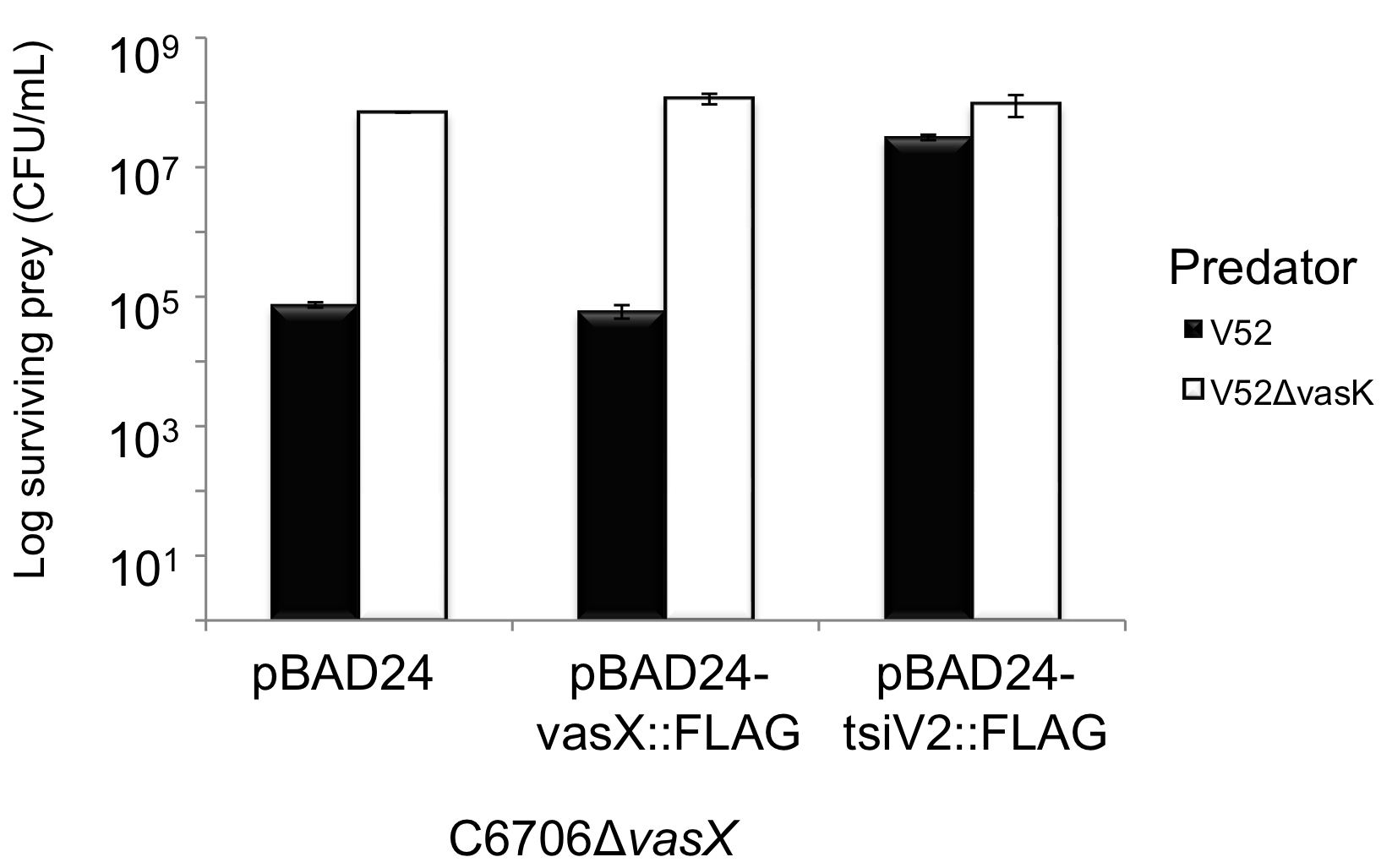

Supplement: Figure S8 — Deletion of vasX renders C6706 sensitive to killing by V52. Survival of rifampicin-resistant C6706ΔvasX and complemented strains (indicated on the x-axis) was determined by measuring CFU following exposure to the indicated rifampicin-sensitive predator listed in the legend. These data are representative of three independent experiments performed in technical duplicate. Error bars indicate the standard deviation. (TIF) [file ppat.1003752.s008.tif]

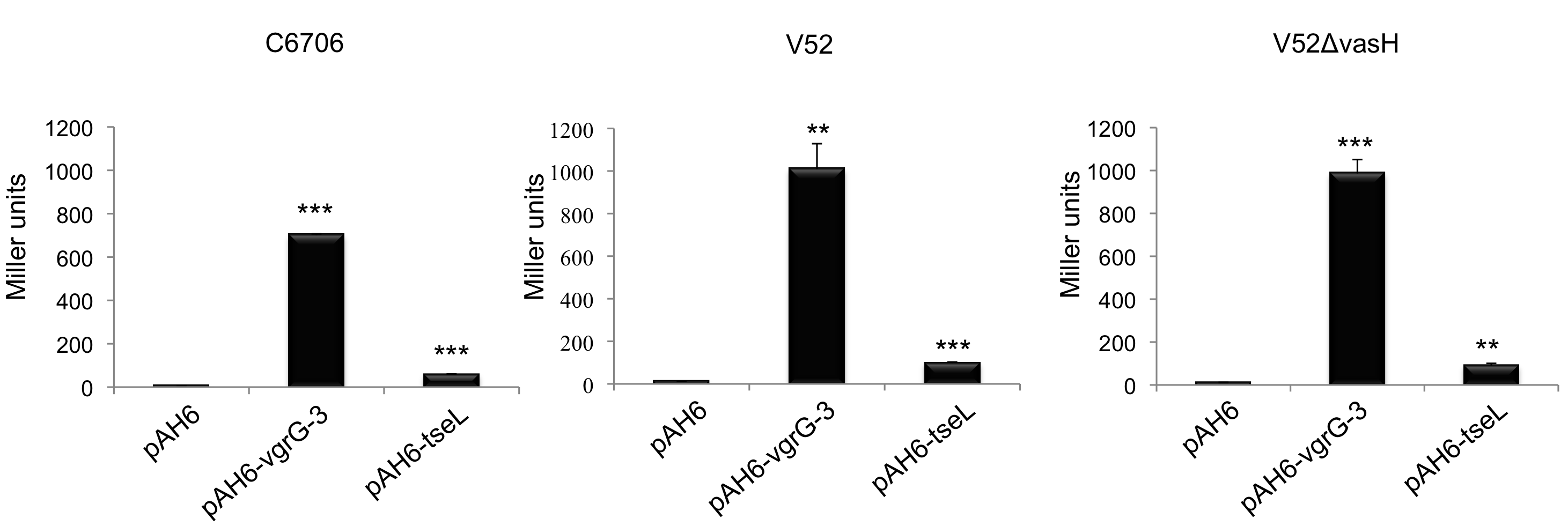

Supplement: Figure S9 — Dual expression profile of tsiV1 and tsiV3. β-galactosidase assays were performed using the strains indicated at the top of each graph that had been transformed with the plasmids indicated on the x-axis. Miller units were calculated and plotted. These data are representative of two independent experiments performed in technical triplicate. Error bars indicate the standard deviation. *** = p<0.001, ** = p<0.005 relative to the empty vector control. p-values were calculated based on the Student's one-tailed, paired t-test. (TIF) [file ppat.1003752.s009.tif]
